# Differential Rac1 signalling by guanine nucleotide exchange factors implicates FLII in regulating Rac1-driven cell migration

**DOI:** 10.1038/ncomms10664

**Published:** 2016-02-18

**Authors:** Hadir Marei, Alejandro Carpy, Anna Woroniuk, Claire Vennin, Gavin White, Paul Timpson, Boris Macek, Angeliki Malliri

**Affiliations:** 1 Cell Signalling Group, Cancer Research UK Manchester Institute, The University of Manchester, Manchester M204BX, UK; 2 Proteome Center Tuebingen, Interfaculty Institute for Cell Biology, University of Tuebingen, Tuebingen 72026, Germany; 3 Invasion and Metastasis Group, Garvan Institute of Medical Research, The Kinghorn Cancer Centre, Faculty of Medicine, St Vincent's Clinical School, University of New South Wales, Darlinghurst, New South Wales 2010, Australia

## Abstract

The small GTPase Rac1 has been implicated in the formation and dissemination of tumours. Upon activation by guanine nucleotide exchange factors (GEFs), Rac1 associates with a variety of proteins in the cell thereby regulating various functions, including cell migration. However, activation of Rac1 can lead to opposing migratory phenotypes raising the possibility of exacerbating tumour progression when targeting Rac1 in a clinical setting. This calls for the identification of factors that influence Rac1-driven cell motility. Here we show that Tiam1 and P-Rex1, two Rac GEFs, promote Rac1 anti- and pro-migratory signalling cascades, respectively, through regulating the Rac1 interactome. In particular, we demonstrate that P-Rex1 stimulates migration through enhancing the interaction between Rac1 and the actin-remodelling protein flightless-1 homologue, to modulate cell contraction in a RhoA-ROCK-independent manner.

## Abstract (web summary)

The small GTPase Rac1 regulates various cellular processes, including cell migration. However, Rac1 can have opposing migratory effects. Here the authors show that two guanine nucleotide exchange factors, Tiam1 and P-Rex1, differentially regulate the Rac1 interactome to determine the downstream phenotype.

## Graphical abstract

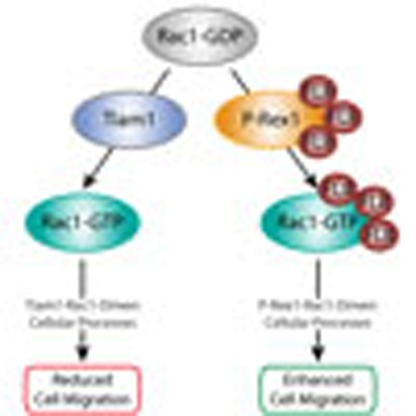

Cell migration and invasion play an important role in various physiological functions, such as embryonic development, immune cell responses and wound healing. In addition, aberrant regulation of cell motility is linked to tumour progression with increased cell migration and invasion marking a key step in metastasis, the major cause of death in cancer patients^1^,^2^. Studies focused on deciphering the underlying mechanisms involved in cell migration and invasion have identified five key steps that govern the mesenchymal-mode of single-cell motility: (1) front-rear polarization; (2) membrane protrusion at the leading edge; (3) cell–extra-cellular matrix (ECM) adhesion; (4) actomyosin contractility; and (5) detachment of the cell rear^3^,^4^. Given the required interplay between the actin cytoskeleton, cell–ECM adhesions and myosin motor contractile forces, various signalling proteins are implicated in regulating cell migration. Rac1, a member of the Rho family of small GTPases, is of particular importance. Similarly to other small GTPases, Rac1 serves as a molecular switch cycling between an inactive GDP-bound form and an active GTP-bound form. Upon activation by guanine nucleotide exchange factors (GEFs), Rac1 undergoes a conformational change allowing it to bind to and activate downstream effectors that directly and indirectly influence cell migration and invasion in normal and cancer cells^3^,^5^,^6^. However, activation of Rac1 can lead to opposing migratory phenotypes. Through its ability to promote front-rear polarization^7^,^8^, lamellipodia formation^9^,^10^,^11^, as well as assembly of focal complexes at the leading edge^12^,^13^, Rac1 promotes cell migration and invasion. However, Rac1 is also essential for maintaining cell–cell contacts in epithelial cells via promoting cadherin-mediated cell–cell junctions^14^,^15^,^16^. As a result, Rac1 is implicated in impeding cell migration and invasion. The dual role of Rac1 in cell migration and invasion is further demonstrated by its ability to regulate the expression and release of matrix metalloproteinases^17^,^18^, which aid in ECM degradation, as well as the matrix metalloproteinase inhibitors, tissue inhibitor of metalloproteinases^19^. These contrasting roles of Rac1 in migration and invasion make its therapeutic potential uncertain and call for the identification of factors that regulate Rac1 downstream specificity.

Rac1 differential downstream effects are often attributed to differences in cell type and upstream signalling from the ECM^20^,^21^; however, the mechanism by which these inputs control selectivity downstream of Rac1 is poorly understood. Interestingly, GEFs have been shown to influence responses downstream of Rho1 in yeast cells^22^,^23^. Moreover, a limited number of studies propose a scaffolding role of GEFs, whereby they bind either directly to GTPase effectors^24^,^25^,^26^ or indirectly through other scaffolding proteins^27^,^28^. Thus GEFs present an interesting class of proteins that could potentially play a role not only in Rac1 activation but also in dictating Rac1 downstream effects that govern its anti-migratory versus pro-migratory cellular phenotypes.

To rigorously evaluate the hypothesis that GEFs determine output downstream of Rac1 by regulating its interaction with effectors, we were particularly interested in comparing Rac1 GEFs known to induce opposing Rac1-driven cellular effects. We therefore focused on Tiam1 and P-Rex1, two Rac GEFs that have been associated with contrasting migratory phenotypes^14^,^29^,^30^,^31^,^32^,^33^. Interestingly, we show that activation of Rac1 by either GEF, under the same cellular conditions, results in distinct morphological phenotypes and differential actin cytoskeletal rearrangements that dictate Rac1 anti- versus pro-migratory roles. In addition, we performed a quantitative mass spectrometry screen uncovering distinct sets of interactors with differential Rac1 binding, dependent on the upstream GEF. Focusing on P-Rex1-enriched Rac1 binding partners, we identify protein flightless-1 homolog (FLII), a gelsolin protein superfamily member, as a novel Rac1 effector that is required for mediating P-Rex1-Rac1-driven cell migration through modulating cell contraction in a RhoA-ROCK-independent manner. Thus, our data demonstrate the importance of GEFs in dictating Rac1 functional specificity through modulating effector binding and uncover a previously unreported signalling cascade that regulates Rac1-driven cell migration.

## Results

### Tiam1 and P-Rex1 induce differential Rac1 downstream effects

**Figure 1:**
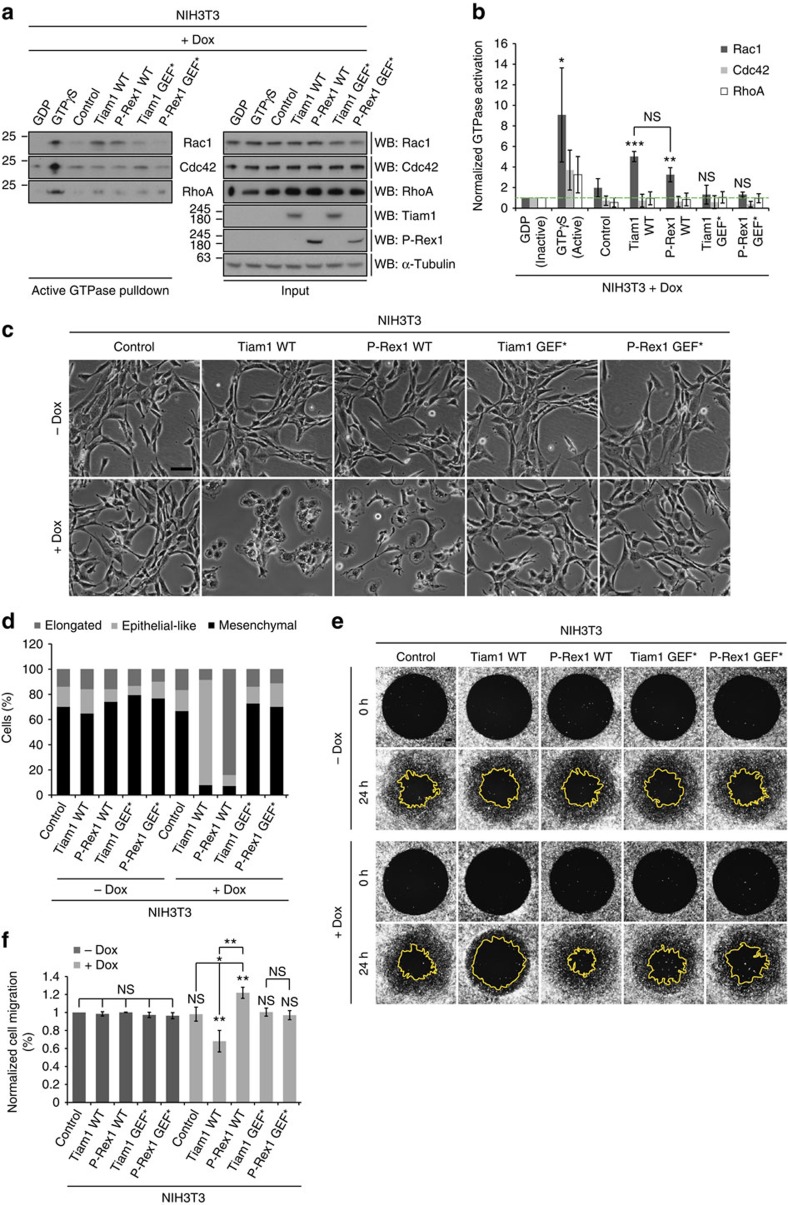
Activation of Rac1 by Tiam1 or P-Rex1 induces differential effects in NIH3T3 cells. (**a**) Lysates from NIH3T3 cells following 1 μg ml^−1^ doxycycline (dox) treatment for 24 h to induce expression of indicated GEF constructs were divided equally and subjected to PAK-CRIB pulldown or Rhotekin pulldown. Levels of active and total Rac1, Cdc42 and RhoA were detected by western blot analysis. GDP and GTPγS loaded NIH3T3 lysates were used as negative and positive pulldown controls, respectively. α-Tubulin was used as a loading control. (**b**) Quantification of levels of active Rac1, Cdc42 and RhoA in NIH3T3 cells described in **a** relative to total levels of the respective proteins normalized to GDP-loaded NIH3T3 lysates±s.e.m. from three independent experiments. The dashed green line indicates basal levels of active GTPases in the GDP control. (**c**) Representative phase-contrast images of NIH3T3 cells treated with ethanol (−dox) or 1 μg ml^−1^ dox (+dox) for 24 h to induce expression of indicated GEF constructs. Scale bar, 100 μm. (**d**) Quantification of GEF-induced cellular phenotypes depicted in **c**. Graph represents per cent cells with the indicated morphology from a total of 150 cells per condition from three independent experiments. (**e**) Representative fluorescence images following Oris migration assay of NIH3T3 cells treated with ethanol (−dox) or 1 μg ml^−1^ dox (+dox) for 24 h to induce expression of indicated GEF constructs. Scale bar, 200 μm. (**f**) Quantification of cell migration of NIH3T3 cells described in **e** normalized to −dox treated control cells. Graph represents the average per cent migration±s.e.m from three independent experiments. In **b**,**f**, student's *t*-test was used to assess significance as indicated on graphs. *P* values indicated above each bar are relative to GDP (**b**) or −dox treated control cells (**f**). NS, non-significant; *=*P*≤0.05; **=*P*≤0.01; ***=*P*≤0.001.

To achieve selective activation of Tiam1-Rac1 and P-Rex1-Rac1 signalling cascades irrespective of upstream signalling, we utilized a doxycycline (dox)-inducible system to overexpress wild-type (WT) Tiam1 or P-Rex1 in NIH3T3 cells. In addition, to exclude phenotypic changes due to GEF overexpression alone rather than GEF-mediated Rac1 activation, previously described Tiam1 and P-Rex1 GEF-dead mutants (GEF*)^34^,^35^ were also introduced into cells and analysed in parallel with WT proteins (Supplementary Fig. 1a). Active Rac1 pulldown experiments demonstrated the ability of Tiam1 WT and P-Rex1 WT but not their GEF* mutants to activate Rac1 (Supplementary Fig. 1b,c). In addition, unlike Rac1, levels of active Cdc42 and RhoA, two small GTPases that are also implicated in cell migration, were not affected upon expression of the different GEF constructs, confirming the selectivity of Tiam1 and P-Rex1 in activating Rac1 (Fig. 1a,b).

Changes in cell morphology and actin cytoskeleton rearrangements are known to accompany cell migration^4^, therefore using the dox-inducible system we examined the effect of GEFs in regulating these processes. In NIH3T3 cells, expression of Tiam1 WT was associated with increased cell aggregation and membrane ruffling inducing an epithelial-like morphology in the otherwise mesenchymal NIH3T3 cells (Fig. 1c,d). These morphological changes were also accompanied with increased actin localization at cell–cell contacts, consistent with the observed cell aggregation (Supplementary Fig. 1d). More importantly, expression of Tiam1 WT resulted in a significant reduction in cell migration (Fig. 1e,f). In contrast, expression of P-Rex1 WT was predominantly associated with an elongated morphology and the formation of thin membrane protrusions (Fig. 1c,d) rich in polymerized actin (Supplementary Fig. 1d). In addition, Oris migration assays demonstrated that unlike Tiam1 WT, P-Rex1 activation of Rac1 resulted in significantly increased cell migration (Fig. 1e,f). Importantly, these observations were limited to dox treated cells expressing the WT proteins but not the GEF* mutants (Fig. 1 and Supplementary Fig. 1), indicating that the observed phenotypes following expression of Tiam1 and P-Rex1 are Rac1-driven.

**Figure 2:**
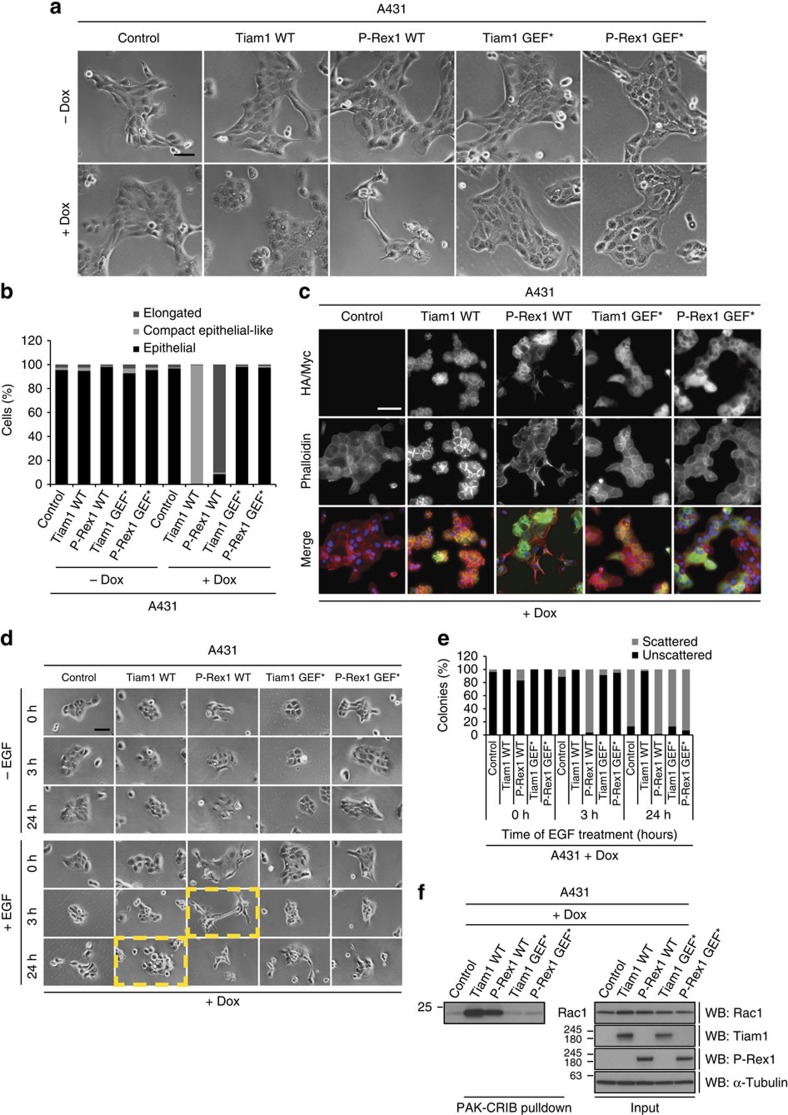
Activation of Rac1 by Tiam1 or P-Rex1 induces differential effects in A431 cells. (**a**) Representative phase-contrast images of A431 cells treated with ethanol (−dox) or 1 μg ml^−1^ doxycycline (+dox) for 24 h to induce expression of indicated GEF constructs. Scale bar, 100 μm. (**b**) Quantification of GEF-induced cellular phenotypes depicted in **a**. Graph represents per cent cells with the indicated morphology from a total of 150 cells per condition from three independent experiments. (**c**) Representative immunofluorescence images of +dox treated A431 cells fixed in 4% formaldehyde. Phalloidin staining was used to visualize the actin cytoskeleton and antibodies against HA-tagged Tiam1 WT and GEF* or Myc-tagged P-Rex1 WT and GEF* to detect the expression of the respective GEFs upon dox induction. DAPI was used to stain the nuclei. Scale bar, 100 μm. (**d**) Representative phase-contrast images of +dox treated A431 cells either left untreated (−EGF) or treated with 100 ng ml^−1^ epidermal growth factor (+EGF) for the indicated times following serum starvation for 18–24 h. Yellow boxes are used to highlight the differential effects of Tiam1 WT and P-Rex1 WT on cell scattering at indicated time points. Scale bar, 100 μm. (**e**) Quantification of cell scattering of+dox treated A431 cells described in **d**. Graph represents per cent of scattered or unscattered colonies from a total of 50 colonies per condition from three independent experiments. (**f**) Lysates from A431 cells following+dox treatment for 24 h to induce expression of indicated GEF constructs were subjected to PAK-CRIB pulldown. Levels of active and total Rac1 were detected by western blot analysis. α-Tubulin was used as a loading control. Representative western blot from three independent experiments.

Interestingly, expression of Tiam1 WT and P-Rex1 WT, but not their GEF* mutants, in the epithelial squamous cell carcinoma cell line, A431, and the epithelial immortalized Madin-Darby canine kidney cell line, MDCKII, resulted in phenotypic effects similar to those observed in NIH3T3 cells (Fig. 2 and Supplementary Fig. 2). Expression of Tiam1 WT induced an even more compact epithelioid morphology associated with membrane ruffling and more defined cell–cell contacts that were distinguishable from control cells (Fig. 2a,b and Supplementary Fig. 2a,b). Consistently, increased actin localization at cell junctions was observed in A431 cells expressing Tiam1 WT (Fig. 2c). On the other hand, P-Rex1 WT expression resulted in a prominent morphological shift to elongated cells (Fig. 2a,b and Supplementary Fig. 2a,b), with disrupted actin staining at cell–cell contacts (Fig. 2c). GEF expression was also accompanied by differential cell migration in MDCKII cells, with Tiam1 WT significantly suppressing migration and P-Rex1 WT promoting migration (Supplementary Fig. 2c,d). Moreover, Tiam1 WT expression impeded epidermal growth factor (EGF)-induced cell scattering in A431 cells and hepatocyte growth factor (HGF)-induced cell scattering in MDCKII cells, while P-Rex1 WT expression resulted in enhanced scattering detected as early as 3 h post growth factor treatment (Fig. 2d,e and Supplementary Fig. 2e,f). As expected and similarly to NIH3T3 cells, elevated levels of active Rac1 were only detected upon expression of the WT but not the GEF* mutants (Fig. 2f and Supplementary Fig. 2g) further confirming the dependency of Tiam1 and P-Rex1 on Rac1 to induce differential phenotypes. Taken together these data demonstrate the ability of Tiam1 and P-Rex1, irrespective of upstream signalling, to induce cell morphological changes and actin cytoskeletal rearrangements that govern Rac1-driven anti- and pro-migratory phenotypes, respectively, in both normal and cancer cell lines.

### Tiam1 and P-Rex1 differentially regulate the Rac1 interactome

Having demonstrated that Tiam1 and P-Rex1 elicit distinct Rac1-driven phenotypes, we were interested in deciphering the mechanism by which these contrasting phenotypes are manifested. We hypothesized that the observed GEF-induced differential cell migratory phenotypes might be mediated by the ability of Tiam1 and P-Rex1 to modulate Rac1-effector binding. To test this hypothesis, we engineered a double dox-inducible system for expression of *StrepII*-FLAG-tagged Rac1 (SF-Rac1) alone or together with either Tiam1 or P-Rex1 (WT or GEF* mutants) in NIH3T3 cells. We then coupled the efficiency of SF-tandem affinity purification (TAP)^36^,^37^,^38^ with the quantitative power of stable isotope labelling by amino acids in cell culture (SILAC)^39^,^40^ to examine the Rac1 interactome following induction of either Tiam1 or P-Rex1. By designing a five-way SILAC screen we were able to directly compare the relative abundance of Rac1-associated proteins upon expression of Tiam1 WT/GEF* or P-Rex1 WT/GEF* to cells expressing SF-Rac1 only (control). Given that the same control cells were used to identify changes in the Rac1 interactome between WT GEF expression (Set1) and GEF* mutant expression (Set2), we were also able to indirectly assess the effect of WT versus GEF* GEF expression on Rac1-effector binding. In addition, to increase the robustness of the SILAC quantification, we performed reverse SILAC in which the SILAC labelling media were switched between Tiam1 WT/GEF* and P-Rex1 WT/GEF* cells (Supplementary Fig. 3a).

Combined, 350 proteins were identified as putative Rac1 interacting proteins from a total of two SILAC and two reverse SILAC screens. We first assessed the ability of the SF-TAP/SILAC approach to identify bona fide Rac1 binding partners via conducting an extensive literature review combined with searching available protein databases, such as the human protein reference database (ref. 41), NetPath (ref. 42) and the human protein–protein interaction predictions database^43^,^44^, to analyse the full list of identified proteins. As expected the SILAC screen yielded several known Rac1 binding partners, predicted Rac1 interactors, as well as proteins implicated in Rac1 signalling (Supplementary Table 1), thus suggesting that a large number of the remaining proteins are genuine novel Rac1 interactors.

From the identified proteins, 231 were associated with quantifiable SILAC ratios in ≥2 SILAC screens. Interestingly, using a commonly accepted cutoff of ±1.3 fold-change^45^, we identified a subset of Rac1 interactors that showed changes in Rac1 binding only upon expression of either GEF (Supplementary Fig. 3b), thus highlighting a role of Tiam1 and P-Rex1 in differentially modulating the Rac1 interactome.

### FLII is a novel P-Rex1-enriched Rac1 binding partner

**Figure 3:**
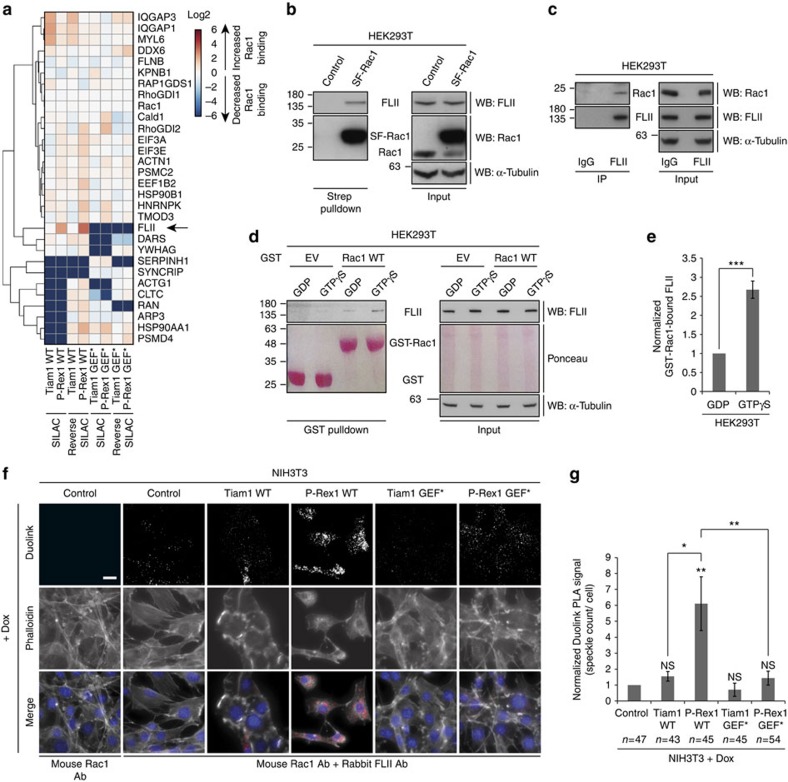
FLII is a novel P-Rex1-enriched Rac1 interactor that binds preferentially to active Rac1. (**a**) Heat map of indicated SILAC identified Rac1 interactors classified under one of the following Ingenuity integrated pathway analysis (IPA) functional groups: cell movement, cell morphology, cell signalling, cell-to-cell signalling and interaction, as well as cell assembly and organization. The heat map represents the Log2 of SILAC protein ratios relative to control cells expressing SF-Rac1 alone from a representative SILAC and reverse SILAC screen. (**b**) Streptavidin (*Strep*) pulldown from HEK293T cells expressing SF-Rac1. Co-precipitated endogenous FLII was detected by western blot analysis. (**c**) Endogenous FLII immunoprecipitation (IP) from HEK293T cells. Co-precipitated endogenous Rac1 was detected by western blot analysis. In **b**,**c**, α-Tubulin was used as a loading control. Representative western blots from three independent experiments. (**d**) GST pulldown using purified GST (EV) or GST-tagged Rac1 WT (Rac1 WT) loaded with GDP or GTPγS and incubated with HEK293T lysates. Co-precipitated endogenous FLII was detected by western blot analysis. α-Tubulin and ponceau staining were used as loading controls. (**e**) Quantification of endogenous GST-Rac1-bound FLII in HEK293T cells described in **d** normalized to GDP-loaded GST-Rac1 WT±s.e.m. from three independent experiments. Student's *t*-test was used to assess significance as indicated on graph. ***=*P*≤0.001. (**f**) Representative immunofluorescence images of NIH3T3 cells fixed in 4% formaldehyde and subjected to the Duolink *in situ* PLA assay following treatment with 1 μg ml^−1^ doxycycline (+dox) for 24 h to induce expression of indicated GEF constructs. Phalloidin and DAPI were used to visualize the actin cytoskeleton and nuclei, respectively. Scale bar, 20 μm. (**g**) Quantification of average Duolink PLA signal from indicated number of NIH3T3 cells described in **f** ±s.e.m. Student's *t*-test was performed to determine statistical significance and *P* values are shown on graph. *P* values indicated above each bar are relative to control cells. NS, non-significant; *=*P*≤0.05; **=*P*≤0.01.

To determine whether GEF-mediated regulation of the Rac1 interactome is important for inducing differential effects downstream of Rac1, we further classified the identified Rac1 binding partners according to their cellular functions using the Ingenuity integrated pathway analysis (IPA) software. Given the observed GEF-induced differential effects on cell migration, we focused on a shortlist of differentially regulated proteins falling within functional categories that relate to cell motility, including cell movement, cell morphology, cell signalling, cell-to-cell signalling and interaction, as well as cell assembly and organization (Fig. 3a). Among the identified proteins, FLII exhibited a significant GEF-dependent Rac1 binding fold-change between Tiam1 WT and P-Rex1 WT expressing cells. Relative to NIH3T3 cells expressing SF-Rac1 alone, SILAC ratios indicated that FLII was associated with approximately a five-fold (*P*=1.46 × 10−6) and a 10-fold (*P*=7.13 × 10−9) increase in SF-Rac1 binding in a forward and reverse SILAC experiment, respectively, that was specific to P-Rex1 WT. In addition, FLII was not co-purified with SF-Rac1 upon expression of either Tiam1 GEF* or P-Rex1 GEF*, suggesting that it is functionally important for P-Rex1-Rac1-driven cellular effects. In contrast, the known Rac1 interactor RhoGDI1 showed limited SILAC ratio fluctuations across the different samples that were comparable to ratios observed for Rac1 itself (Supplementary Fig. 3c), indicating that the relative abundance of RhoGDI1 protein associated with Rac1 does not change upon expression of the different GEF constructs. Taken together, these data demonstrate that SILAC ratios associated with proteins identified from the screens are not due to fluctuations in Rac1 purification, and more importantly that expression of P-Rex1 WT induces specific changes in FLII SILAC ratios that are not observed for other proteins, such as RhoGDI1.

FLII is a member of the actin-remodelling gelsolin protein superfamily and has been implicated in cell migration. It has been shown to translocate from the nucleus and perinuclear region in motile NIH3T3 cells to actin-rich regions at the cell periphery, including membrane ruffles and the leading edge^46^. Moreover, FLII is implicated in regulating focal adhesion turnover through inhibiting the phosphorylation of paxillin in a Rac1-dependent manner^47^, providing a link between FLII and Rac1 signalling. Using western blot analysis, we confirmed that exogenous SF-Rac1 co-precipitates endogenous FLII following a streptavidin (*Strep*) pulldown of SF-Rac1 from HEK293T cells (Fig. 3b). In addition, endogenous FLII also co-precipitated endogenous Rac1 confirming that FLII is a novel Rac1 binding partner (Fig. 3c). Furthermore, GST-pulldown experiments with purified GST or GST-Rac1 loaded with either GDP or GTPγS and incubated with endogenous FLII from HEK293T lysates revealed that FLII binds preferentially to active Rac1 (Fig. 3d,e).

To further validate this interaction, we utilized the Duolink *in situ* proximity ligation assay (PLA) to visualize the interaction between endogenous Rac1 and endogenous FLII upon expression of the different GEFs in NIH3T3 cells. Interestingly, an increased Duolink signal was only detected upon expression of P-Rex1 WT (Fig. 3f,g). We also investigated the effect of P-Rex1 WT overexpression in MCF7 cells, which express higher levels of endogenous P-Rex1 compared with NIH3T3 cells, thus presenting a cell line that is configured to respond physiologically to P-Rex1 signalling. Similarly to NIH3T3 cells, overexpression of P-Rex1 WT in MCF7 cells stimulated the endogenous Rac1-FLII interaction as indicated by the increased Duolink signal detected only in P-Rex1 WT expressing cells (Supplementary Fig. 3d,e). Pulldown of SF-Rac1 from MCF7 cells following SF-TAP also revealed an enhanced interaction with endogenous FLII upon expression of P-Rex1 WT but not the other GEF constructs. In contrast, there was no change in the levels of co-precipitated RhoGDI1 upon expression of the different GEF constructs (Supplementary Fig. 3f,g). Together, the Duolink *in situ* PLA assays and the SF-TAP further validate the SILAC screen and confirm that the Rac1-FLII interaction is enhanced upon P-Rex1 WT expression.

### FLII is a novel P-Rex1 binding partner

**Figure 4:**
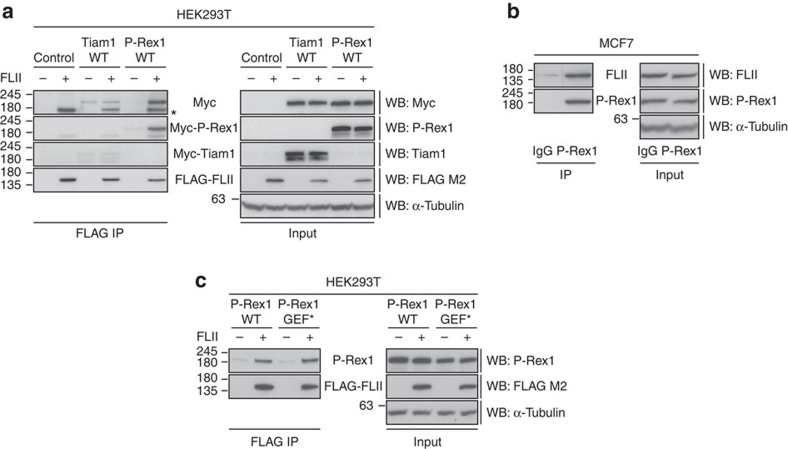
FLII is a novel P-Rex1 interactor that does not bind to Tiam1. (**a**) FLAG immunoprecipitation (IP) from HEK293T cells expressing Myc-tagged Tiam1 WT or Myc-tagged P-Rex1 WT alone or together with FLAG-tagged FLII. Co-precipitated Myc-tagged GEFs were detected by western blot analysis. * indicates background band. (**b**) Endogenous P-Rex1 IP from MCF7 cells. Co-precipitated endogenous FLII was detected by western blot analysis. (**c**) FLAG IP from HEK293T cells expressing Myc-tagged P-Rex1 WT or GEF* alone or together with FLAG-tagged FLII. Co-precipitated Myc-tagged GEFs were detected by western blot analysis. In **a**–**c**, α-Tubulin was used as a loading control. Representative western blots from three independent experiments.

Given the proposed role of GEFs in controlling GTPase signalling through acting as molecular scaffolds, we were interested in determining whether, in addition to Rac1, FLII binds to P-Rex1 and not Tiam1. Immunoprecipitation of FLAG-tagged FLII from HEK293T cells co-expressing Myc-Tiam1 WT or Myc-P-Rex1 WT revealed that FLII binds preferentially to P-Rex1 (Fig. 4a). Similarly, Myc-P-Rex1 WT but not Myc-Tiam1 WT co-precipitated FLAG-tagged FLII (Supplementary Fig. 4a). The P-Rex1-FLII interaction was also confirmed on an endogenous level using MCF7 cells following immunoprecipitation of endogenous P-Rex1 (Fig. 4b) or endogenous FLII (Supplementary Fig. 4b). P-Rex1 has been shown to drive cell migration and invasion in a number of melanoma cell lines, including CHL1 (ref. 32). Thus, to address whether this interaction occurs in a cellular setting in which P-Rex1 promotes migration and invasion we utilized the Duolink *in situ* PLA assay to visualize P-Rex1-FLII binding in CHL1 cells. Interestingly, there was a significant increase in the Duolink signal in CHL1 cells when both the P-Rex1 and FLII antibodies were applied compared with cells subjected to the FLII antibody only, indicating a strong endogenous interaction in these cells (Supplementary Fig. 4c,d). Moreover, we found that both P-Rex1 WT and P-Rex1 GEF* bind to FLII in equal levels, demonstrating that the P-Rex1-FLII interaction is independent of the P-Rex1 GEF activity (Fig. 4c) potentially occurring upstream of Rac1 activation. Although GEF activity is not a prerequisite for P-Rex1-FLII interaction, GTP-loading of Rac1 enhances the Rac1 interaction with FLII. This hints at a dual role of P-Rex1 as both a Rac1 GEF and a molecular scaffold.

### FLII binds to Rac1 and P-Rex1 through different domains

**Figure 5:**
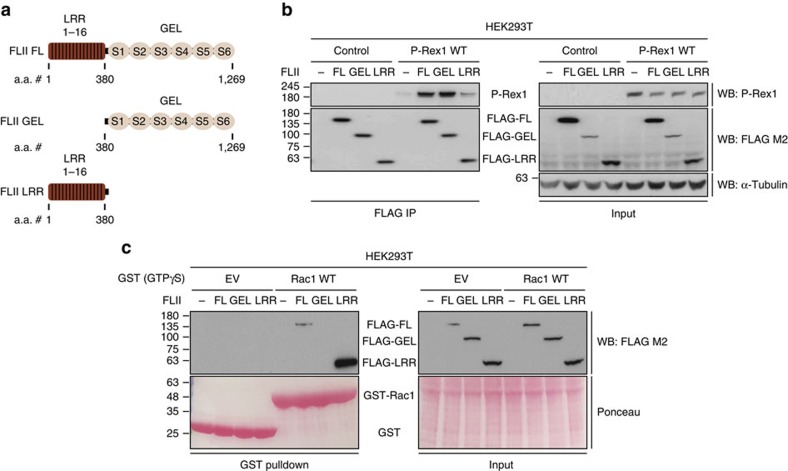
P-Rex1 and Rac1 bind preferentially to the GEL and LRR domain of FLII, respectively. (**a**) Schematic representation of FLII domain structure showing 16 leucine-rich repeats (LRR) comprising the N-terminal LRR domain and six gelsolin-like repeats (S1-S6) representing the C-terminal GEL domain together with the amino acid number (a.a. #) range for each domain. FLII FL, full-length FLII; FLII GEL, gelsolin domain only; FLII LRR, LRR domain only. (**b**) FLAG immunoprecipitation (IP) from HEK293T cells expressing the different FLAG-tagged FLII domain mutants outlined in **a** alone or together with P-Rex1 WT. Co-precipitated exogenous P-Rex1 was detected by western blot analysis. α-Tubulin was used as a loading control. Representative western blot from three independent experiments. (**c**) GST pulldown using purified GST (EV) or GST-tagged Rac1 WT (Rac1 WT) loaded with GTPγS and incubated with HEK293T lysates expressing the different FLAG-tagged FLII domain mutants outlined in **a**. Co-precipitated FLAG-tagged FLII domain mutants were detected by western blot analysis. Ponceau staining was used as a loading control. Representative western blot from three independent experiments.

As a member of the gelsolin protein superfamily, FLII possesses the characteristic gelsolin-like (GEL) domain. However, unlike other superfamily members, FLII also contains an N-terminal leucine-rich repeats (LRR) domain (Fig. 5a) with the two domains governing distinct functions. The GEL domain is mainly responsible for FLII-mediated actin remodelling^48^. On the other hand, LRR domains serve as protein–protein interaction motifs via forming a doughnut- or horseshoe-like conformation that serves as a hydrophobic pocket for protein binding^49^,^50^,^51^. To gain functional insight into the Rac1-FLII and P-Rex1-FLII interactions, previously described FLAG-tagged full-length FLII (FLII FL), a GEL only mutant (FLII GEL), and an LRR only mutant (FLII LRR) (ref. 52) (Fig. 5a) were used to determine the FLII domain responsible for mediating its interaction with Rac1 and P-Rex1. Intriguingly, FLAG co-immunoprecipitation of the different FLII domain mutants revealed that P-Rex1 binds to the GEL domain (Fig. 5b), whereas Rac1 binds preferentially to the LRR domain of FLII (Supplementary Fig. 5a). Using purified proteins we showed that P-Rex1 binds directly to the GEL domain of FLII (Supplementary Fig. 5b). Rac1 binding to the LRR domain of FLII was also further confirmed by GST pulldown of purified GTPγS loaded GST-Rac1 incubated with lysates from HEK293T cells expressing the different FLAG-tagged FLII domain mutants (Fig. 5c). Together, the above data imply that P-Rex1, Rac1 and FLII might form a ternary complex in cells that is functionally important for eliciting P-Rex1-Rac1-driven cellular phenotypes.

### FLII is required for P-Rex1-Rac1-driven cell migration

**Figure 6:**
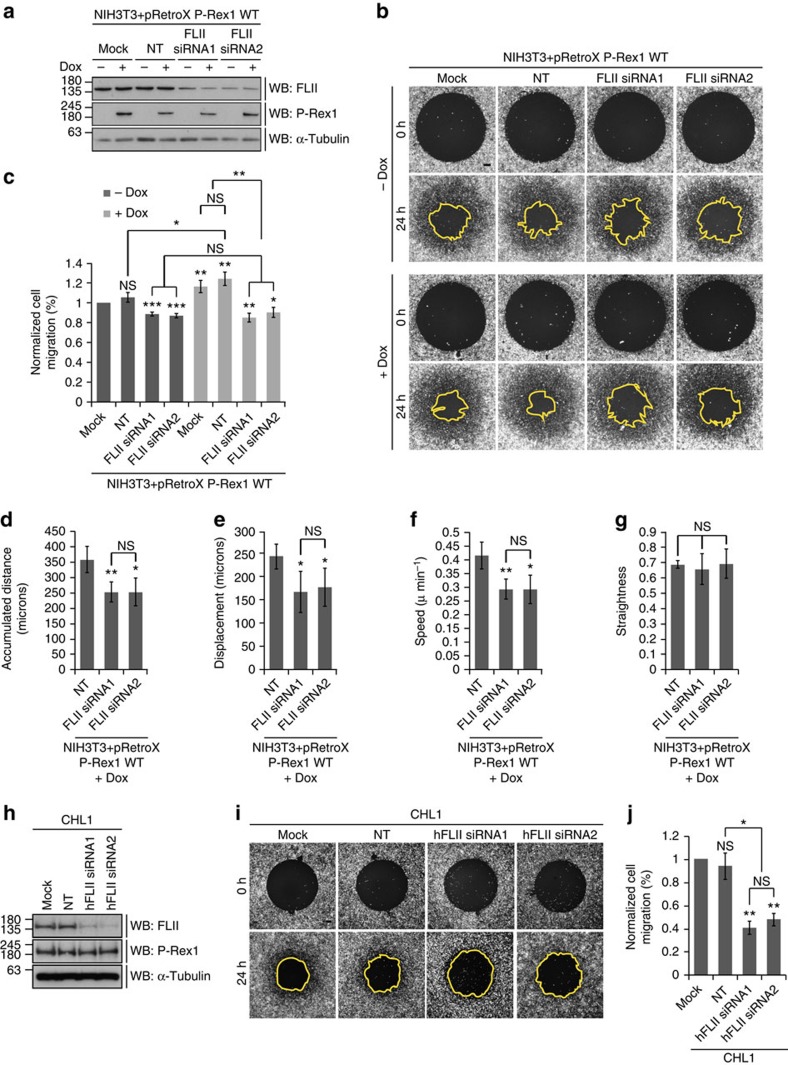
FLII is required for P-Rex1-Rac1-driven cell migration. (**a**) Western blot of NIH3T3 cells (NIH3T3+pRetroX P-Rex1 WT) treated with mock, NT or two different siRNAs against FLII (FLII siRNA1 and FLII siRNA2) in the presence of ethanol (−dox) or 1 μg ml^−1^ doxycycline (+dox) to induce expression of P-Rex1 WT. Levels of endogenous FLII and P-Rex1 expression were detected by western blot analysis. (**b**) Representative fluorescence images following Oris migration assay of cells described in **a**. Scale bar, 200 μm. (**c**) Quantification of cell migration of NIH3T3 cells described in **a** normalized to −dox treated mock cells. (**d**–**g**) Single-cell tracking of NIH3T3 cells expressing P-Rex1 WT following dox induction and treatment with indicated oligos. Graphs show the effect of FLII knockdown on (**d**) accumulated distance, (**e**) displacement, (**f**) speed and (**g**) straightness. (**h**) Western blot of CHL1 cells transfected with NT oligo or two different siRNAs against human FLII (hFLII siRNA1 and hFLII siRNA2). Levels of endogenous FLII were detected by western blot analysis. In **a**,**h** α-Tubulin was used as a loading control. Representative western blots from three independent experiments. (**i**) Representative fluorescence images following Oris migration assay of CHL1 cells treated with indicated oligos. Scale bar, 200 μm. (**j**) Quantification of cell migration of CHL1 cells described in **h** normalized to mock treated cells. For **c**,**j** graphs represent the average per cent migration±s.e.m. from three independent experiments. For **c**–**g**,**j**, Student's *t*-test was used to assess significance as indicated on graphs. *P* values indicated above each bar are relative to −dox treated mock cells (**c**), or NT control (**d**–**g**) or mock treated cells (**j**). NS=non-significant; *=*P*≤0.05; **=*P*≤0.01; ***=*P*≤0.001.

Despite a negative role for FLII in cell migration having previously been described^53^,^54^,^55^,^56^, P-Rex1 promotes migration through Rac1 activation concomitant with increased Rac1-FLII binding, raising the possibility that FLII might positively influence migration. FLII depletion by siRNA in both −dox and+dox treated control NIH3T3 cells was associated with a modest yet significant decrease in the ability of cells to migrate when compared with mock and non-targeting (NT) treated cells (Supplementary Fig. 6a). Similar results were observed in −dox treated NIH3T3 cells transduced with the different GEF dox-inducible systems (Supplementary Fig. 6b–d and Fig. 6a–c). Interestingly, compared with mock and NT treated cells, there was no additive effect of FLII knockdown on the ability of Tiam1 WT to reduce cell migration in NIH3T3 cells (Supplementary Fig. 6b). FLII depletion in Tiam1 GEF* and P-Rex1 GEF* expressing cells, on the other hand, resulted in a modest yet significant reduction in cell migration (Supplementary Fig. 6c,d) similar to that observed in control NIH3T3 cells (Supplementary Fig. 6a). Importantly, FLII depletion was sufficient to abolish the ability of P-Rex1 WT to stimulate cell migration (Fig. 6a–c).

To further understand the role of FLII in P-Rex1-Rac1-driven cell migration we performed single-cell tracking following scratch assays in NIH3T3 cells treated with dox to induce expression of P-Rex1 WT. Consistent with the ORIS migration assays described above, FLII depletion using two different siRNAs was associated with a significant decrease in cell migration as measured by various parameters, including accumulated distance, cell displacement and speed (Fig. 6d–f). Interestingly, FLII knockdown had no effect on the straightness of the track of cell movement (Fig. 6g) indicating that while FLII is important for mediating P-Rex1-driven cell migration it does not influence directionality. Active Rac1 pulldown experiments following FLII depletion in NIH3T3 cells expressing P-Rex1 WT also demonstrated that the diminished migratory capacity of FLII knockdown cells is not due to reduced levels of either total or active Rac1 in these cells (Supplementary Fig. 6e,f). Importantly, levels of active RhoA were not affected upon expression of P-Rex1 WT in confluent monolayers of NIH3T3 cells that were scratched to induce cell migration (Supplementary Fig. 6g,h). Similarly, there was no significant increase in the levels of active RhoG in NIH3T3 cells expressing P-Rex1 WT upon stimulation of migration following scratching (Supplementary Fig. 6i) suggesting that P-Rex1 acts directly on Rac1 and not through RhoA or RhoG to stimulate cell migration in a FLII-dependent manner.

Knockdown of FLII was also associated with reduced cell migration in CHL1 cells (Fig. 6h–j), indicating that FLII is important for optimal cell migration in a cellular setting in which endogenous P-Rex1 is known to drive cell migration and invasion^32^. Interestingly, in migrating CHL1 cells both P-Rex1 and FLII co-localize at the leading edge together with actin (Supplementary Fig. 6j). All together, these data highlight the interplay between P-Rex1, Rac1 and FLII that is required for optimal P-Rex1-Rac1-driven cell migration.

### FLII is required for P-Rex1-induced cell contractility

**Figure 7:**
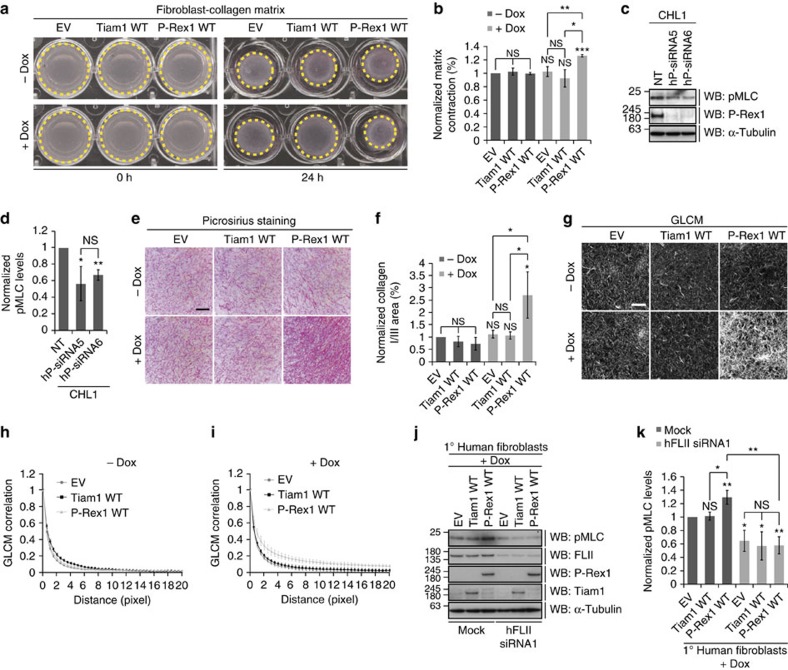
FLII is required for P-Rex1-driven cell contraction. (**a**) Fibroblast-collagen matrices were treated with ethanol (−dox) or 1 μg ml^−1^ doxycycline (+dox) to induce expression of indicated constructs and scans were taken at 0 and 24 h post treatment. (**b**) Quantification of cell contraction depicted in **a** normalized to −dox treated EV control. Graph represents the average per cent matrix contraction±s.e.m. from three independent experiments. (**c**) CHL1 cells were transfected with NT, or two different siRNAs against human P-Rex1 (hP-siRNA5, hP-siRNA6). Levels of endogenous pMLC and P-Rex1 were detected by western blot analysis. (**d**) Quantification of pMLC levels in CHL1 cells described in **c** normalized to α-Tubulin and pMLC levels detected in the NT control. Graph represents average pMLC levels±s.e.m. from three independent experiments. (**e**) Collagen picrosirius staining of matrices described in **a**. Scale bar, 50 μm. (**f**) Quantification of picrosirius staining depicted in **e** normalized to −dox treated EV control. Graph represents the area covered by collagen I and III±s.e.m. from three independent experiments. (**g**) Representative GLCM images of matrices described in **a**. Scale bar, 100 μm. (**h**,**i**) GLCM correlation decay curves of matrices described in **a** showing −dox (**h**) and+dox (**i**) curves. (**j**) 1° human fibroblasts were either mock transfected or treated with siRNA against human FLII (hFLII siRNA1) in the presence of dox to induce expression of indicated constructs. Levels of endogenous pMLC and FLII were detected by western blot analysis. For **c**,**j**, α-Tubulin was used as a loading control. (**k**) Quantification of pMLC levels in 1° human fibroblasts described in **j** normalized to α-Tubulin and pMLC levels detected in the EV control. Graph represents average pMLC levels±s.e.m. from three independent experiments. For **b**,**d**,**f**,**k**, Student's *t*-test was used to assess significance as indicated on graphs. *P* values indicated above each bar are relative to −dox treated EV control (**b**,**f**), NT control (**d**) or mock treated EV control (**k**). NS, non-significant; *=*P*≤0.05; **=*P*≤0.01; ***=*P*≤0.001.

FLII has been shown to regulate cell contraction^47^. Activation of myosin II through myosin light chain (MLC) phosphorylation induces actomyosin contractility that is crucial for mediating cell migration^4^. The level of phosphorylated myosin II at the cell centre and rear is mediated through RhoA-ROCK signalling^57^; however, two recent studies have implicated Rac1 in regulating myosin II phosphorylation and localization at the leading edge^58^,^59^. This combined with our findings demonstrating that GEF expression does not induce RhoA activation in NIH3T3 cells hinted at a potential role of P-Rex1 in promoting contractility in a FLII-dependent manner. To explore this possibility, primary (1°) human fibroblasts expressing pRetroX-Tight-Pur empty vector (EV), Tiam1 WT or P-Rex1 WT were mixed with acid extracted rat-tail collagen I and the ability of either GEF to mediate contraction was assessed using a fibroblast-collagen matrix contraction assay as previously described^60^. Interestingly, expression of P-Rex1 WT, but not Tiam1 WT enhanced fibroblast-collagen matrix contraction significantly compared with the −dox treated and EV controls (Fig. 7a,b). This was further demonstrated by examining the levels of phosphorylated MLC (pMLC) upon expression of either GEF. We found that expression of P-Rex1 WT but not Tiam1 WT resulted in a significant increase in levels of pMLC compared with the EV control (Supplementary Fig. 7a,b). Consistently, immunohistochemistry of fibroblast-collagen matrices following expression of the different constructs showed a significant increase in the number of pMLC positive cells, only upon expression of P-Rex1 WT (Supplementary Fig. 7c). Importantly, depletion of endogenous P-Rex1 in both CHL1 and MCF7 cells was associated with a significant reduction in pMLC levels (Fig. 7c,d and Supplementary Fig. 7d,e). Consistent with the increased contractile nature of P-Rex1 WT expressing fibroblasts, only fibroblast-collagen matrices prepared using P-Rex1 WT expressing 1° human fibroblasts exhibited increased collagen content and crosslinking as indicated by picrosirius staining and second harmonic generation (SHG) imaging, respectively (Fig. 7e,f and Supplementary Fig. 7f,g). Additionally, we utilized gray-level co-occurrence matrix (GLCM) analysis^61^ on fibroblast-collagen matrices following 9 days of GEF expression to examine changes in collagen remodelling. As suggested by the picrosirius staining and SHG imaging, expression of P-Rex1 WT but not Tiam1 WT was associated with increased collagen crosslinking and remodelling (Fig. 7g–i). We next examined the dependency of P-Rex1-mediated cell contraction on FLII. Interestingly, FLII depletion by siRNA in 1° human fibroblasts was associated with an overall reduction in pMLC levels regardless of which construct was expressed. More importantly, FLII knockdown in P-Rex1 WT expressing cells inhibited P-Rex1 WT-induced MLC phosphorylation (Fig. 7j,k). This data highlights a FLII-dependent role of P-Rex1 in regulating cell contraction, thus uncovering a novel signalling cascade by which P-Rex1 can mediate Rac1-driven cell migration through FLII.

### Cell contraction regulates P-Rex1-driven cell migration

**Figure 8:**
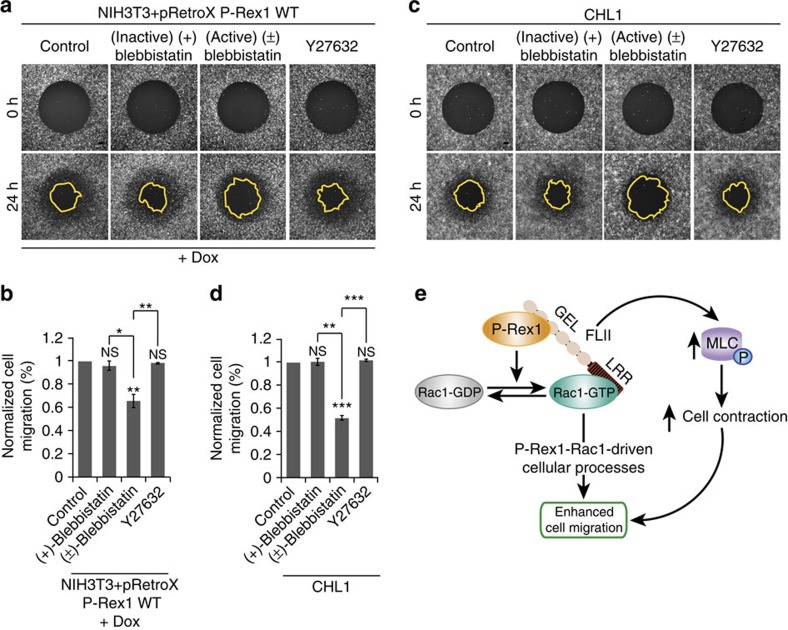
RhoA-ROCK-independent cell contraction is required for P-Rex1-Rac1-FLII-driven cell migration. (**a**) Representative fluorescence images following Oris migration assay of NIH3T3 cells either left untreated (control), or treated with 100 μM inactive enantiomer of blebbistatin [(+) blebbistatin], 100 μM blebbistatin [(±) blebbistatin] or 10 μM ROCK inhibitor (Y27632) in the presence of 1 μg ml^−1^ doxycycline (+dox) to induce expression of P-Rex1 WT. Scale bar, 200 μm. (**b**) Quantification of cell migration of NIH3T3 cells described in **a** normalized to control cells. (**c**) Representative fluorescence images following Oris migration assay of CHL1 cells either left untreated (control) or treated with indicated chemicals. Scale bar, 200 μm. (**d**) Quantification of cell migration of CHL1 described in **c** normalized to control cells. For **b**,**d**, graphs represent the average per cent migration±s.e.m. from three independent experiments. Student's *t*-test was used to assess significance as indicated on graphs. *P* values indicated above each bar are relative to control cells. NS, non-significant; *=*P*≤0.05; **=*P*≤0.01; ***=*P*≤0.001. (**e**) Schematic representation of the P-Rex1-Rac1-FLII signalling cascade. Activation of Rac1 by P-Rex1 results in Rac1 binding to a set of downstream effectors, including FLII. P-Rex1, via its scaffolding ability, binds to FLII through its GEL domain and brings it in close proximity to active Rac1 further stimulating the Rac1-FLII interaction via the LRR domain of FLII. Through this interaction, P-Rex1 induces phosphorylation of MLC (pMLC) thereby enhancing cell contraction in a FLII-dependent manner. This cascade accounts, in part, for P-Rex1-Rac1-driven cell migration.

To further explore the role of cell contractility in P-Rex1-Rac1-FLII-driven cell migration we performed ORIS migration assays using NIH3T3 cells expressing P-Rex1 WT upon dox induction that were either left untreated (control) or treated with an inactive enantiomer of blebbistatin [(+)-blebbistatin], blebbistatin [(±)-blebbistatin], or a ROCK inhibitor (Y27632). Interestingly, blebbistatin treatment was associated with a significant reduction in P-Rex1-driven cell migration, while the ROCK inhibitor had no effect on cell migration (Fig. 8a,b). Similarly, treatment of CHL1 cells with blebbistatin but not the inactive enantiomer or the ROCK inhibitor impeded migration (Fig. 8c,d), indicating that RhoA-ROCK-independent cell contraction is required for optimal P-Rex1-driven cell migration in an endogenous setting. Taken together, these results indicate that P-Rex1 promotes cell contractility and consequently migration through Rac1 and FLII in a RhoA-ROCK-independent manner.

## Discussion

When considering the contribution of Rac1 signalling to cancer dissemination, a challenge facing the field is accounting for its contrasting effects on migration and invasion. This conundrum can be resolved by defining contexts eliciting a given behaviour and identifying factors that dictate Rac1 downstream signalling cascades that govern its anti- and pro-migratory roles. Work presented here provides direct evidence implicating GEFs in modulating Rac1 downstream signalling. Through the use of a controlled cell system in which GEF expression is the only variable, we demonstrate that expression of Tiam1 and P-Rex1 induces distinct phenotypes in three different cell lines. In addition, via assessing the role of GEF* mutants of both proteins, we show that these effects are Rac1-driven. Moreover, the quantitative proteomic screen we undertook has allowed us to begin to dissect the mechanisms by which GEFs regulate the Rac1 interactome to mediate Rac1 anti- and pro-migratory effects. This is demonstrated by the role we now show for FLII in regulating P-Rex1-driven cell contraction, which accounts, at least in part, for P-Rex1-Rac1-mediated cell migration (Fig. 8e).

Through our work, we confirm that Tiam1 activation of Rac1 stimulates cell–cell adhesion and reduces cell migration. This was particularly evident in the mesenchymal NIH3T3 cells in which we show that expression of Tiam1 induces an epithelial-like morphology accompanied by reduced cell migration, as previously described by Sander *et al.*^29^ A similar phenotypic switch is also seen in metastatic melanoma cells, in which Tiam1 is shown to impede cell migration through regulating the actin cytoskeleton to promote cell–cell contacts and induce an epithelial-like morphology^30^. In addition, we demonstrate that Tiam1 not only antagonizes HGF-induced cell scattering of MDCKII cells, as reported by Hordijk *et al.*^14^, but also impedes EGF-induced scattering of A431 cells. Together these results suggest that Tiam1 activation of Rac1 is associated with reduced cell migration potentially through its ability to increase actin and cadherin deposition at cell–cell contacts, as previously suggested^14^,^30^. The selective ability of Tiam1 to promote Rac1-dependent anti-migratory functions may be through regulation of the Rac1 interactome. Indeed, our proteomic analysis indicates an increased binding of Rac1 and IQGAP1 upon Tiam1 expression. Interestingly, active Rac1 has been shown to reduce the ability of IQGAP1 to interact with β-catenin^62^, thereby counteracting the negative regulatory role of IQGAP1 on cadherin-mediated cell–cell adhesions^63^. In addition, active Rac1 suppresses IQGAP1-induced translocation of α-catenin which precedes cell scattering in MDCKII cells^64^. The increased Rac1-IQGAP1 binding upon Tiam1 expression might therefore explain the anti-migratory phenotypic changes associated with these cells.

In contrast to Tiam1, P-Rex1 expression in NIH3T3, A431 and MDCKII cells induces the formation of actin-rich membrane protrusions and cells exhibit reduced cell–cell contacts with an increased rate of cell migration and scattering. Consistent with our observations, P-Rex1-Rac1 signalling has been shown to drive cell migration in an immortalized primary human fibroblast cell line^31^. *In vivo* studies also show that mice deficient in P-Rex1 have a decreased rate of melanoblast migration^32^. In addition, upregulation of P-Rex1 has been implicated in promoting metastasis in prostate cancer as well as in melanoma^32^,^33^. Importantly, our data describes a novel signalling cascade involving P-Rex1, Rac1 and FLII that promotes cell migration. Evidence from the literature supports a crosstalk between FLII and small GTPases, where FLII has been shown to co-localize with Ras and other small GTPases at the leading edge of motile cells in actin-rich structures, including membrane ruffles and lamellipodia^46^. FLII was shown to interact with Ras *in vitro* through the LRR domain^65^. We show that similarly to Ras, Rac1 binds to the LRR domain of FLII. In contrast, P-Rex1 binds to the GEL domain of FLII. These observations suggest that both P-Rex1 and Rac1 could bind to FLII simultaneously forming a ternary complex that mediates P-Rex1-Rac1-driven cellular functions (Fig. 8e).

Intriguingly, FLII is considered a negative regulator of wound healing and cell migration^47^,^53^,^54^,^55^,^56^,^66^. However, we show that FLII is required for P-Rex1-Rac1-driven cell migration through modulating P-Rex1-mediated cell contraction. This indicates that, similarly to Rac1, FLII plays a dual role in regulating cell migration that is potentially dependent on the cellular context and FLII binding partners. Consistent with our data, fibroblasts from FLII heterozygous (FLII^+/−^) mice exhibit reduced contractility in collagen contraction assays^47^. Activation of myosin II through the phosphorylation of MLC has been directly implicated in cell migration through its role in promoting stress fibre formation and focal adhesion assembly in the centre of migrating cells. However, this process is mainly mediated through RhoA and its downstream effector ROCK^57^. Given that expression of P-Rex1 WT does not affect levels of active RhoA in migrating cells, together with the inability of ROCK inhibition to impede P-Rex1-driven cell migration, our findings support a RhoA-ROCK-independent pathway by which MLC phosphorylation and cell contraction can be modulated in cells, potentially at the leading edge where we observed co-localization of P-Rex1 and FLII. This is particularly interesting since Rac1 has been shown to regulate chemotaxis through its effector PAK1, which modulates the phosphorylation and localization of myosin IIB at the leading edge^58^. More recently, Rac1 activation has also been shown to stimulate MLC phosphorylation through inhibiting the MLC phosphatase^59^. Our data also indicates that P-Rex1-mediated cell contraction is important for collagen crosslinking and remodelling. We propose that P-Rex1-induced collagen remodelling is also dependent on FLII since FLII has recently been shown to interact with nonmuscle myosin IIA (NMMIIA) at cell protrusions, which is important for cell extension formation that enables FLII-dependent collagen remodelling by fibroblasts^67^. Given the link between ECM remodelling and cancer metastasis (reviewed in ref. 68), the role of P-Rex1 in collagen crosslinking and remodelling described in this manuscript may have important implications for P-Rex1-Rac1-driven cancer cell invasion and metastasis.

In conclusion, our data provides clear evidence supporting a role of Tiam1 and P-Rex1 in driving differential signalling cascades downstream of Rac1 through modulating the Rac1 interactome. Characterization of the P-Rex1-FLII and Rac1-FLII interactions also highlights a novel role of P-Rex1 as a scaffolding protein to stimulate Rac1 pro-migratory phenotypes. Taken together our data indicate that P-Rex1 not only activates Rac1 but is also able to direct proteins, such as FLII, to the active form of Rac1 to mediate specific cellular functions, including cell contraction, that enable P-Rex1 to enhance cell migration. Therefore, this study provides insight into previously unreported P-Rex1-Rac1 cellular functions that mediate cell migration and highlights additional modes by which P-Rex1 and Rac1 might drive cancer dissemination.

## Methods

### Antibodies

Details of primary and secondary antibodies used in this study are summarized in Supplementary Table 2.

### Cell culture

All cell lines were cultured at 37 °C and 5% CO_2_ using DMEM (Life Technologies, 61965-026) supplemented with 10% v/v tetracycline-free fetal bovine serum (Tet-free FBS; Labtech International Ltd, FB-1001T/500) and 10 μg ml^−1^ penicillin-streptomycin (Life Technologies, 15140-122). Low glucose DMEM (1 g l^−1^; Life Technologies, 10567-014) was used for MDCKII cells. All experiments were conducted in full serum media supplemented with penicillin-streptomycin unless otherwise indicated. NIH3T3, A431, MDCKII, HEK293T, MCF7, CHL1 were obtained from ECACC, operated by Public Health England or ATCC. Primary human fibroblasts, described by Timpson *et al.*^69^ were used for this study.

### Constructs

Details of plasmids used in this study are summarized in Supplementary Table 3.

### Generation of cell lines

Transfection with the indicated constructs was performed using the *Trans*IT-LT1 reagent (Mirus, MIR 2305) according to manufacturer's instructions. Retroviral transduction was performed by transfection of Phoenix packaging cells with retroviral constructs. Polybrene (10 μg ml^−1^; Sigma-Aldrich, 107689) was added to the cleared supernatant collected 24 h post transfection and the mixture was used to transduce cell lines. This was repeated three times with 24 h intervals using fresh supernatant each time. For antibiotic selection, cells transduced with the pRetroX-Tet-On Advanced plasmid only (control cells) were grown in tetracycline-free DMEM media with 1 mg ml^−1^ G418 selection (Sigma-Aldrich, A1720). Cells with both the pRetroX-Tet-On Advanced and pRetroX-Tight-Pur (EV or with indicated proteins) were selected using 1 mg ml^−1^ G418 and 2 μg ml^−1^ Puromycin (Sigma-Aldrich, P8833). NIH3T3 cells expressing histone-2B-GFP were subjected to 5 μg ml^−1^ blasticidin antibiotic selection (Sigma-Aldrich, 15205). Antibiotic selection was withdrawn during experiments. For induction of protein expression cells were treated with 1 μg ml^−1^ doxycycline (+dox) for 24–48 h before analysis. As a dox treatment control, cells were treated with an equal volume of 100% ethanol (−dox).

### Transient transfection of siRNA

For knockdown of mouse FLII in NIH3T3 cells before ORIS migration assay or single-cell tracking, two FLII siRNA oligonucleotides (20 μM stock; Sigma-Aldrich), outlined below, were prepared using the DharmaFECT 1 transfection reagent (GE Healthcare, T-2001-01) or the Lipofectamine RNAiMAX (Life Technologies, 137781) according to manufacturer's instructions and cells were reverse transfected. Cells were re-transfected 48 h later in the presence of ethanol (−dox) or 1 μg ml^−1^ dox (+dox) for an additional 24 h. To determine the efficiency of siRNA transfection, levels of FLII were determined by western blot analysis.

FLII siRNA 1 (Mm01_00187916) 5′-CAGAUCAACUACAAGCUCU[dT][dT]-3′

FLII siRNA 2 (Mm01_00187920) 5′-GACUUUGAUGGGCUGCCUU[dT][dT]-3′

Transient silencing of human FLII in primary (1°) human fibroblasts and CHL1 cells and of human P-Rex1 in CHL1 and MCF7 cells was achieved by reverse transfection of the siRNA oligonucleotides, detailed below, from either Eurofins MWG operon or Qiagen using Lipofectamine RNAiMAX according to manufacturer's instructions. Western blot analysis was used to detect levels of FLII or P-Rex1 48 h post transfection.

hFLII siRNA1 5′-GCUGGAACACUUGUCUGUG[dT][dT]-3′

hFLII siRNA2 5′CAACCUGACCACGCUUCAU[dT][dT]-3′

hP-siRNA5 Hs_P-Rex1_5 FlexiTube siRNA (Qiagen, 1027418-SI03144449)

5′-CGAGUGUAACAGCAAUCGA[dT][dT]-3′

hP-siRNA6 Hs_P-Rex1_6 FlexiTube siRNA (Qiagen, 1027418-SI03246383)

5′-GGGUCAGCCCACCCUUCAA[dT][dT]-3′

Transfection reagent alone (mock) or together with Dharmacon NT siRNA #1 (GE Healthcare, D-001210-01-20) or siRNA#4 (GE Healthcare, D-001210-04-20), were used as controls.

### Analysis of cell morphology

Cells were seeded in the presence of ethanol (−dox) or 1 μg ml^−1^ dox (+dox) for 24 h and phase-contrast images were taken. Cells were classified into three groups based on their morphology as outlined in Supplementary Methods.

### Cell scattering assays

Cells were seeded at a concentration of 2 × 10^3^ cells per well in a six-well plate for 24 h in DMEM media. Cells were then washed twice with phosphate-buffered saline (PBS; 137 mM NaCl, 2.7 mM KCl, 10 mM Na_2_PO_4_, 2 mM KH_2_PO_4_ in dH_2_O) to remove any traces of serum followed by culturing in serum-free media (DMEM+0.01% FBS) for 18–24 h before the addition of HGF (10 ng ml^−1^) or EGF (100 ng ml^−1^). Phase-contrast images of individual colonies were captured at 0, 3 and 24 h post growth factor addition and used for quantification as outlined in Supplementary Methods.

### ORIS migration assay

Cells were plated at a concentration of 5 × 10^5^ cells per well in a six-well plate in the presence of ethanol (−dox) or 1 μg ml^−1^ dox (+dox) 24 h before assay. Cells were trypsinised, counted, and ≈5 × 10^5^ cells were then incubated with Dic16 dye (2.5 μM) for 30 min at 37 °C and 5% CO_2_. Dic16-labelled cells (5 × 10^4^ cells per well) were seeded on fibronectin (5 μg ml^−1^; Sigma-Aldrich, F1141) coated 96-well plates fitted with stoppers (Platypus Technologies, CMA1.101) together with ethanol (−dox) or 1 μg ml^−1^ dox (+dox). Cells were incubated overnight in a humidity chamber at 37 °C and 5% CO_2_ before the removal of the stoppers. For analysis of cell migration following inhibition of cell contraction, cells were incubated with DMEM+10% Tet-free FBS alone (control), or supplemented with the inactive enantiomer of blebbistatin [(+)-blebbistatin] (100 μM; Merk Millipore, 203392), blebbistatin [(±)-blebbistatin] (100 μM; Merk Millipore, 203390) or the ROCK inhibitor Y27632 (10 μM; Sigma-Aldrich, Y0503) for 24 h post stopper removal. Fluorescence images were taken using the low light microscope system (× 4 and × 5 magnification) at 0 and 24 h upon removal of stoppers and cell migration was quantified as outlined in Supplementary Methods.

### Single-cell tracking

NIH3T3 cells expressing histone-2B-GFP were generated through reverse transfection with pBOS-Histone-2B-GFP. Cells were plated at a density of 5 × 10^4^ cells per well into optically clear, flat-bottomed 96-well plates to achieve confluency. Confluent monolayers were wounded with a sterile 10 μl pipette tip and 10% Tet-free FBS supplemented DMEM was replaced, with thorough PBS washes before and after wounding. Images were captured and analysed as outlined in Supplementary Methods.

### Fibroblast-collagen matrix contraction assay

1° human fibroblasts were transiently co-transduced with the respective pRetroX EV/GEF together with pRetroX-Tet-On Advanced to generate a transient dox-inducible system for expression of EV or the indicated GEF constructs. Fibroblast-collagen matrices were prepared as previously described by Timpson *et al.*^69^ with minor modifications. In brief, virally transduced cells were mixed with collagen I (2 mg ml^−1^) in 3 ml 10 × MEM and ≈3 ml 0.22 M NaOH to achieve an alkaline mixture. The fibroblast-collagen mix (500 μl per well) was added gently to 24-well plates and left to set at 37 °C, 5% CO_2_ before adding media containing ethanol (−dox) or 1 μg ml^−1^ dox (+dox) for 24 h to induce protein expression. Quantification of cell contraction was performed as outlined in Supplementary Methods.

### Biochemical analysis of cell contraction

Transiently transduced 1° human fibroblasts were plated at sub-confluency and 24 h later were treated with ethanol (−dox) or 1 μg ml^−1^ dox (+dox) for 24 h. Cells were lysed in IP lysis buffer [50 mM Tris-HCL pH 7.5, 150 mM NaCl, 1% Triton-X-100 (v/v), 10% glycerol (v/v), 2 mM EDTA, 25 mM NaF, 2 mM NaH_2_PO_4_, 1% protease inhibitor cocktail (Sigma-Aldrich, P8340) (v/v), 1% phosphatase inhibitor cocktails 1 and 2 (Sigma-Aldrich, P5726, P0044) (v/v) in dH_2_O] and lysates were mixed with equal volume of 2 × SDS–polyacrylamide gel electrophoresis (PAGE) sample buffer [50% NuPAGE LDS sample buffer 4 × (Life Technologies, NP0008) (v/v), 20% NuPAGE sample reducing agent 10 × (Life Technologies, NP0004) (v/v) in dH_2_O]. Levels of phosphorylated MLC (pMLC) were detected by western blot analysis. For analysis of pMLC in FLII depleted cells, hFLII siRNA1 was added to cells 24 h post plating for an additional 24 h. Protein expression was then induced by dox for 24 h after which cells were lysed and analysed by western blot analysis. Western blot analysis was conducted 48 h post transfection with hP-siRNA5 and hP-siRNA6 to analyse effect of P-Rex1 depletion on pMLC.

### Histological analysis

Samples were fixed in 4% paraformaldehyde overnight before paraffin embedding. Histological staining was performed on 4 and 20 μm sections deparaffinised in xylene and rehydrated using graded ethanol washes. Hematoxylin and eosin staining and counterstaining were undertaken ona Leica autostainer containing Hematoxylin reagent (Thermo Scientific, ASHB1000737AQ) and Eosin Y solution with Phloxine (Sigma-Aldrich, HT110332). A picrosirius red stain kit (Polysciences, 24901-250) was used for picrosirius staining. Mouse anti-pMLC (Ser19) (1:200; Cell Signaling, 3675) was used for pMLC immunohistochemistry staining.

### Second harmonic generation (SHG) imaging of fibrillar collagen I

Hematoxylin and eosin paraffin sections (20 μm) of fibroblast-collagen matrices were used for SHG imaging of fibrillar collagen. Images were captured and analysed as outlined in Supplementary Methods.

### Gray-level co-occurrence matrix (GLCM) analysis

Stromal collagen fibre organization and crosslinking in fibroblast-collagen matrices was assessed using GLCM analysis. This method allows the study of the texture of a sample and provides a readout of the level of crosslinked collagen organization, as previously described^60^,^61^,^70^. GLCM analysis was performed in ImageJ software as outlined in Supplementary Methods.

### Immunofluorescence

Cells grown on coverslips in the presence of ethanol (−dox) or 1 μg ml^−1^ dox (+dox) for 24 h were fixed in 4% formaldehyde for 15 min and incubated for 3 min with cell permeabilization buffer (0.5% Triton-X (v/v) in PBS) at room temperature. Cells were next blocked in 1% bovine serum albumin (BSA; Roche, 10735078001) in PBS for 30 min before incubation with relevant primary antibodies (in 1% BSA) for 1 hour at room temperature. The respective fluorophore-conjugated secondary antibodies were then added for an additional hour at room temperature in the dark. Antibodies used for immunofluorescence are outlined in Supplementary Table 2. Coverslips were mounted onto slides using the ProLong Gold antifade reagent with DAPI stain (Invitogen, P36935) and images were captured using the low light microscope system and processed with Metamorph software (Molecular Devices). Images shown were analysed in ImageJ. Additional details on the specifications of microscopes used for image recording are described in Supplementary Methods.

### Duolink *in situ* proximity ligation assay

Cells seeded on glass coverslips in the presence of 1 μg ml^−1^ dox for 24 h were fixed in 4% formaldehyde and subjected to Duolink *in situ* PLA using mouse anti-Rac1 antibody (1:100; BD Biosciences, 610650) and rabbit anti-FLII antibody (1:100; Sigma-Aldrich, HPA007084), the respective Duolink *in situ* PLA probes (Olink Bioscience, anti-mouse 92004-0100, anti-rabbit 92002-0100) and the Duolink *in situ* detection reagent kit (Olink Bioscience, 92013-0100, 92014-0100) according to manufacturer's instructions. For CHL1 cells, mouse anti-FLII antibody (1:100; Santa Cruz, sc-21716) and rabbit anti-P-Rex1 antibody (1:100; Sigma-Aldrich, HPA001927) were used for the Duolink *in situ* PLA analysis. Coverslips were mounted onto slides using ProLong Gold antifade reagent with DAPI stain (Life Technologies, P36935) and images were taken using the low light microscope system (× 100 magnification). Phalloidin and DAPI were used as fluorescence markers against the actin cytoskeleton and the nucleus, respectively. Quantification of the Duolink signal was performed as outlined in Supplementary Methods.

### Active GTPase pulldown

NIH3T3 cells lysed in GST lysis buffer [25 mM Tris-HCL pH 7.2, 150 mM NaCl, 5 mM MgCl_2_, 1% Nonidet P40 (v/v), 5% glycerol (v/v), 1% protease inhibitor cocktail (v/v), 1% phosphatase inhibitor cocktails 1 and 2 (v/v) in dH_2_O] were divided equally and subjected to either biotinylated PAK-CRIB pulldown or GST-Rhotekin pulldown to detect levels of active Rac1/Cdc42 and RhoA, respectively. For measuring Rac1 and Cdc42 activity, lysates were incubated with 60 μl *Strep*-Tactin superflow resin (IBA GmbH, 2-1206-10) together with 6 μg biotinylated PAK-CRIB purified peptide for 1 hour at 4 °C. For measuring RhoA activity the active RhoA pulldown and detection kit (Thermo Scientific, 16116) was used following manufacturer's instructions. GDP and GTPγ loaded NIH3T3 lysates were used as negative and positive active GTPase pulldown controls, respectively. Levels of active and total Rac1, Cdc42 and RhoA were detected by western blot analysis using the respective primary antibodies. To detect levels of active RhoA in migrating cells, P-Rex1 WT expressing NIH3T3 cells were plated in confluent monolayers that were subjected to multiple scratches to stimulate migration. Lysates were prepared 24 h post scratching and dox induction and analysed using the active RhoA pulldown and detection kit.

### Active RhoG ELISA

NIH3T3 cells were plated in confluent monolayers that were subjected to multiple scratches to stimulate migration. Lysates were prepared 24 h post scratching and dox induction and analysed using the RhoG ELISA kit (Biosource, MBS9324983) according to manufacturer's instructions.

### GST-pulldown

GST or GST-Rac1 WT immobilized on Glutathione Sepharose 4B (GE Healthcare, 17-0756-01) were washed using GST lysis buffer followed by nucleotide chelating using 0.5 M EDTA (10 mM) and incubation with Guanosine 5′-diphosphate sodium salt (GDP; 100 μM; Sigma-Aldrich, G7127) or Guanosine 5′-O-(3-thiotriphosphate) tetralithium salt (GTPγS; 1 mM; Sigma-Aldrich, G8634) for 15 min at 30 °C. Termination of the reaction was achieved by adding 1 M MgCl_2_ (60 mM). HEK293T cells were lysed in GST lysis buffer and equal protein amounts were incubated with GDP or GTPγS loaded GST and GST-Rac1 WT beads for 2 h at 4 °C. Beads were washed following incubation and eluted using 20 μl 2 × SDS–PAGE sample buffer for western blot analysis.

### *In vitro* protein interaction

GST or GST-FLII GEL immobilized on Glutathione Sepharose 4B were washed using GST lysis buffer before incubation with 1 μg recombinant P-Rex1 full-length protein for 2 h at 4 °C. Beads were washed following incubation and eluted using 20 μl 2x SDS–PAGE sample buffer for western blot analysis.

### Immunoprecipitation and pulldowns

Cells were lysed in GST lysis buffer or SF-TAP lysis buffer [30 mM Tris pH 7.4, 150 mM NaCl, 0.5% Nonidet P40 (v/v), 1% protease inhibitor cocktail (v/v), 1% phosphatase inhibitor cocktails 1 and 2 (v/v) in dH_2_O]. For immunoprecipitation, lysates were incubated with 20–50 μl antibody pre-bound to GammaBind G Sepharose beads (GE Healthcare, 17-0885-01) for 1–2 h at 4 °C, washed and resuspended in 20–25 μl of 2x SDS–PAGE sample buffer for western blot analysis. For *Strep* pulldown, lysates were incubated with 200 μl *Strep*-Tactin superflow resin for 90 min at 4 °C, washed and then eluted by incubating with 500 μl desthiobiotin elution buffer (2.5 mM; Sigma-Aldrich, D1411) for 30 min at 4 °C. For FLAG immunoprecipitation, lysates were incubated with 50 μl anti-FLAG M2 affinity gel (Sigma-Aldrich, A2220) for 75 min at 4 °C, washed and then eluted using the 3xFLAG peptide (200 μg ml^−1^; Sigma-Aldrich, F4799). Amicon filter units with a 3 kDa protein cutoff (EMD Millipore, UFC500396) were used to concentrate the *Strep*-Tactin superflow and FLAG eluates. Concentrated protein samples were mixed with 20 μl 2 × SDS–PAGE sample buffer for western blot analysis. Details of antibodies and commercial conjugated beads used for immunoprecipitation and pulldowns in this study are listed in Supplementary Table 2.

### *StrepII*-FLAG tandem affinity purification

The *StrepII*-FLAG (SF-TAP) was performed as outlined by Gloeckner *et al.*^36^ with minor modifications. In brief, cells transduced with the dox-inducible system for expressing SF-Rac1 alone or together with the indicated GEFs were treated with either ethanol (−dox) or 1 μg ml^−1^ dox (+dox) and incubated for 48 h. Cells were then lysed in SF-TAP lysis buffer and equal protein amounts were subjected to a *Strep* pulldown as detailed above. Eluates were then subjected to FLAG immunoprecipitation as outlined earlier. Amicon filter units with a 3 kDa protein cutoff were used to concentrate the FLAG eluates. Concentrated protein samples were mixed with 20 μl 2 × SDS–PAGE sample buffer and analysed by western blot analysis or stored at −80 °C for later use.

### Western blot analysis

Samples were prepared as indicated in the respective experimental sections and were resolved using NuPAGE Novex Tris-Acetate pre-cast gels (Life Technologies, 3–8% 10-well EA0375BOX, 3–8% 12-well EA03752BOX) or NuPAGE Novex Bis-Tris pre-cast gels (Life Technologies, 12% 10-well NP0341BOX, 12% 12-well NP0342BOX, 4–12% 10-well NP0321BOX, 12-well NP0321BOX). The BLUeye Pre-Stained Protein Ladder (Geneflow Ltd, S6-0024) was run alongside the samples for protein size reference. Proteins were transferred from gels onto Immobolin PVDF membranes (Millipore, IPFL00010). Western blot analysis was performed using antibodies listed in Supplementary Table 2 as outlined therein and visualized on Hyperfilm ECL (GE Healthcare, 28–9068) using the ECL western blotting analysis system (GE Healthcare, RPN2109). Adobe Photoshop software was used to crop full blots (Supplementary Figs 8 and 9). Band intensities were quantified using the ImageJ Gel analysis tool.

### SILAC labelling and protein identification

Stable isotope labelling by amino acids in cell culture (SILAC) media was prepared using high glucose DMEM without lysine (K) or arginine (R) (PAA) supplemented with 10% dialysed FBS (Life Technologies, 26400-044), 1% L-glutamine (Sigma-Aldrich, G7513) and 10 μg ml^−1^ penicillin-streptomycin. For labelling, cells were cultured for six doubling rounds using three different SILAC culturing media prepared using different amino acids stock solutions as outlined in Supplementary Tables 4 and 5. The efficiency of SILAC labelling was evaluated by determining the amino acid incorporation rate as outlined in Supplementary Methods. For protein identification by mass spectrometry, concentrated mixed SF-TAP eluates were prepared and processed as detailed in Supplementary Methods.

### Ingenuity analysis and heat map generation

Information obtained from the SILAC screens were used to generate a heat map representing a cluster of Rac1 binding partners falling under one of the following IPA functional groups: cell movement, cell morphology, cell signalling, cell-to-cell signalling and interaction, as well as cell assembly and organization. The heat map, generated using the R Project software, represents the Log2 of SILAC protein ratios relative to control cells expressing SF-Rac1 alone from a representative SILAC and reverse SILAC experiment.

### Statistical analysis

For assessing statistical significance between two sets of data a two-tailed paired or two-sample equal variance student's *t*-test was conducted with *P* value ≤0.05 considered significant and *P* value ≤0.01 considered highly significant. Test and *P* values are specified in figure legends where appropriate.

## Additional information

**How to cite this article:** Marei, H. *et al.* Differential Rac1 signalling by guanine nucleotide exchange factors implicates FLII in regulating Rac1-driven cell migration. *Nat. Commun.* 7:10664 doi: 10.1038/ncomms10664 (2016).

## Supplementary Material

Supplementary Information Supplementary Figures 1-9, Supplementary Tables 1-5, Supplementary Methods and Supplementary References
