# Supplementary material for: Differential Rac1 signalling by guanine nucleotide exchange factors implicates FLII in regulating Rac1-driven cell migration
Source: Nat Commun. 2016 Feb 18;7:10664. doi: 10.1038/ncomms10664 (PMC4759627; doi:10.1038/ncomms10664)
Supplement: Supplementary Information — Supplementary Figures 1-9, Supplementary Tables 1-5, Supplementary Methods and Supplementary References [file ncomms10664-s1.pdf]

Supplementary Figure 1

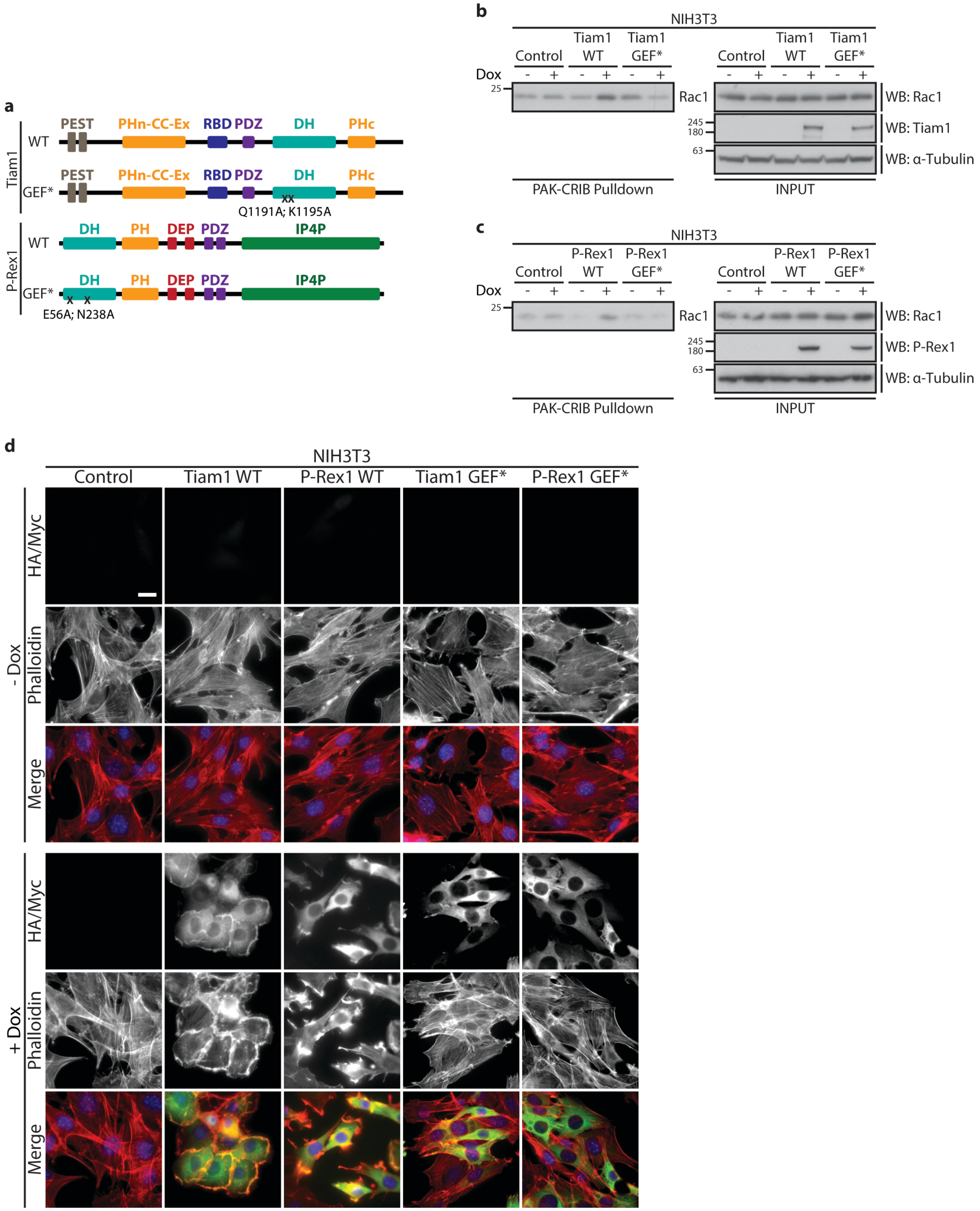

Supplementary Figure 1. Expression of *wild Type* Tiam1 and P-Rex1 but not GEF-dead mutants result in increased levels of active Rac1 and induce distinct actin cytoskeletal rearrangements in NIH3T3 cells. (a) Schematic representations of the domain structures of Tiam1 wild type (WT), Tiam1 GEF-dead mutant (GEF*), P-Rex1 WT and P-Rex1 GEF*. PH= Pleckstrin homology domain; PHn= N-terminal PH domain; PHc= C-terminal PH domain; CC= Coiled-coil domain; EX= Extended structure; RBD= Ras binding domain; PDZ= PSD-95/Dlg/ZO-1 domain; DH= Dbl homology domain; DEP= Dishevelled, Egl-10 and Pleckstrin domain; IP4P= Inositol polyphosphate 4-phosphatase. **(b, c)** Lysates from NIH3T3 cells following ethanol (- dox) or 1 $\mu g\ ml^{-1}$ doxycycline (+ dox) treatment for 24 hours to induce expression of indicated GEF constructs were subjected to PAK-CRIB pulldown. Levels of active and total Rac1 were detected by Western blot analysis. α-Tubulin was used as a loading control. Representative Western blots from three independent experiments. **(d)** Representative immunofluorescence images of – dox and + dox treated NIH3T3 cells fixed in 4 % formaldehyde. Phalloidin staining was used to visualise the actin cytoskeleton and antibodies against HA-tagged Tiam1 WT and GEF* or Myc-tagged P-Rex1 WT and GEF* were used to detect the expression of the respective GEFs upon dox induction. DAPI was used to stain the nuclei. Scale bar= 20 μm.

Supplementary Figure 2

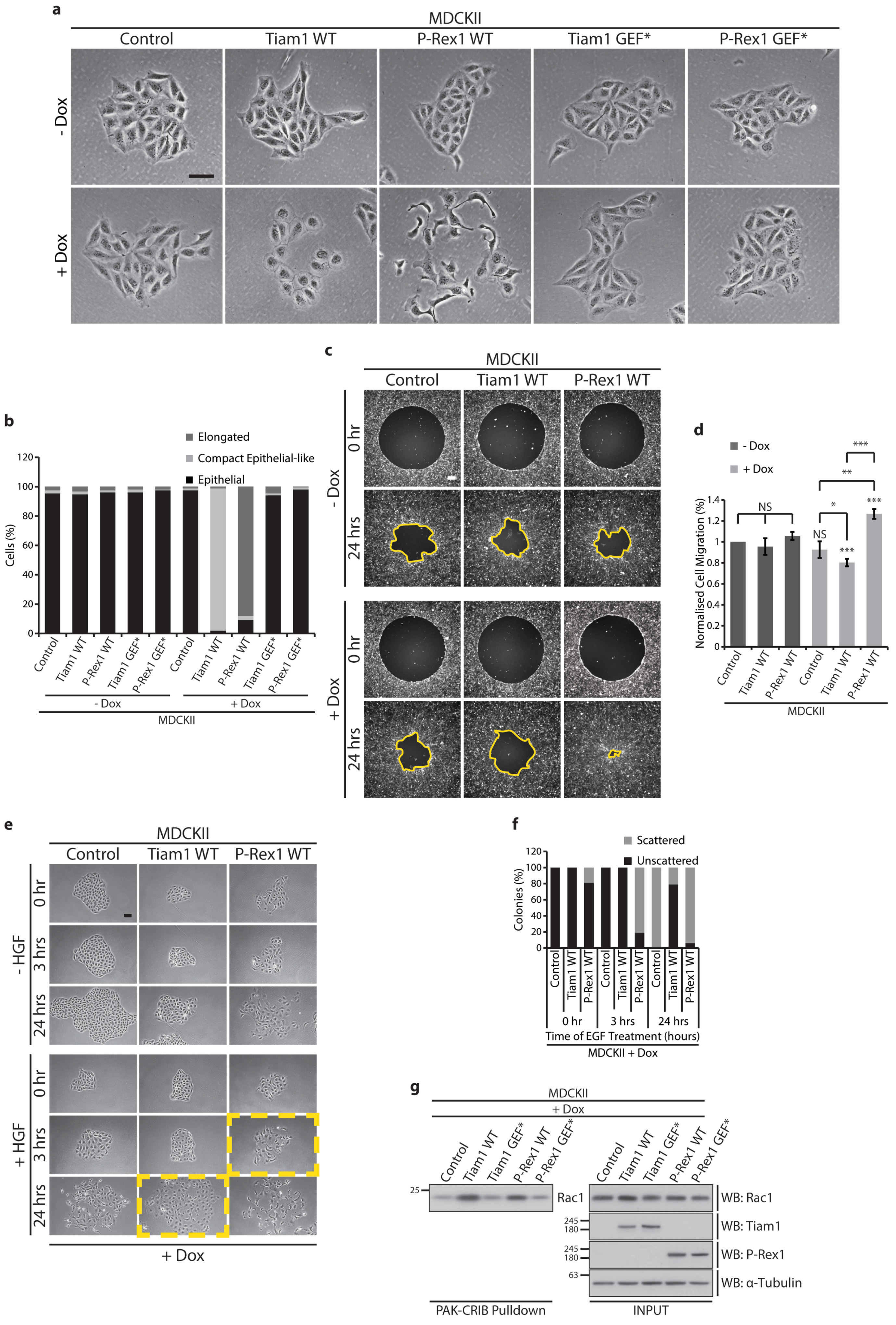

Supplementary Figure 2. Activation of Rac1 by Tiam1 or P-Rex1 induces differential effects in MDCKII cells. (a) Representative phase-contrast images of MDCKII cells treated with ethanol (- dox) or 1 μg ml $^{-1}$ doxycycline (+ dox) for 24 hours to induce expression of indicated GEF constructs. Scale bar= 100 μm. **(b)** Quantification of GEF-induced cellular phenotypes depicted in (a). Graph represents percent cells with the indicated morphology from a total of 150 cells per GEF construct from three independent experiments. **(c)** Representative fluorescence images following Oris™ migration assay of MDCKII cells treated with ethanol (- dox) or 1 μg ml $^{-1}$ dox (+ dox) for 24 hours to induce expression of indicated GEF constructs. Scale bar= 200 μm. **(d)** Quantification of cell migration of MDCKII cells described in (c) normalised to - dox treated control cells. Graph represents the average percent migration ± s.e.m. from three independent experiments. Student's t-test was used to assess significance as indicated on graph. p-values indicated above each bar are relative to – dox treated control cells. NS= non-significant; *= $p \leq 0.05$; **= $p \leq 0.01$; ***= $p \leq 0.001$. **(e)** Representative phase-contrast images of + dox treated MDCKII cells either left untreated (- HGF) or treated with 10 ng ml $^{-1}$ Hepatocyte Growth Factor (+ HGF) for the indicated times following serum starvation for 18-24 hours. Yellow boxes are used to highlight the differential effects of Tiam1 WT and P-Rex1 WT on cell scattering at indicated time points. Scale bar= 100 μm. **(f)** Quantification of cell scattering of + dox treated MDCKII cells described in (e). Graph represents percent of scattered or unscattered colonies from a total of 50 colonies per GEF construct from three independent experiments. **(g)** Lysates from MDCKII cells following + dox treatment for 24 hours to induce expression of indicated GEF constructs were subjected to PAK-CRIB pulldown. Levels of active and total Rac1 were detected by Western blot analysis. α-Tubulin was used as a loading control. Representative Western blot from three independent experiments.

Supplementary Figure 3

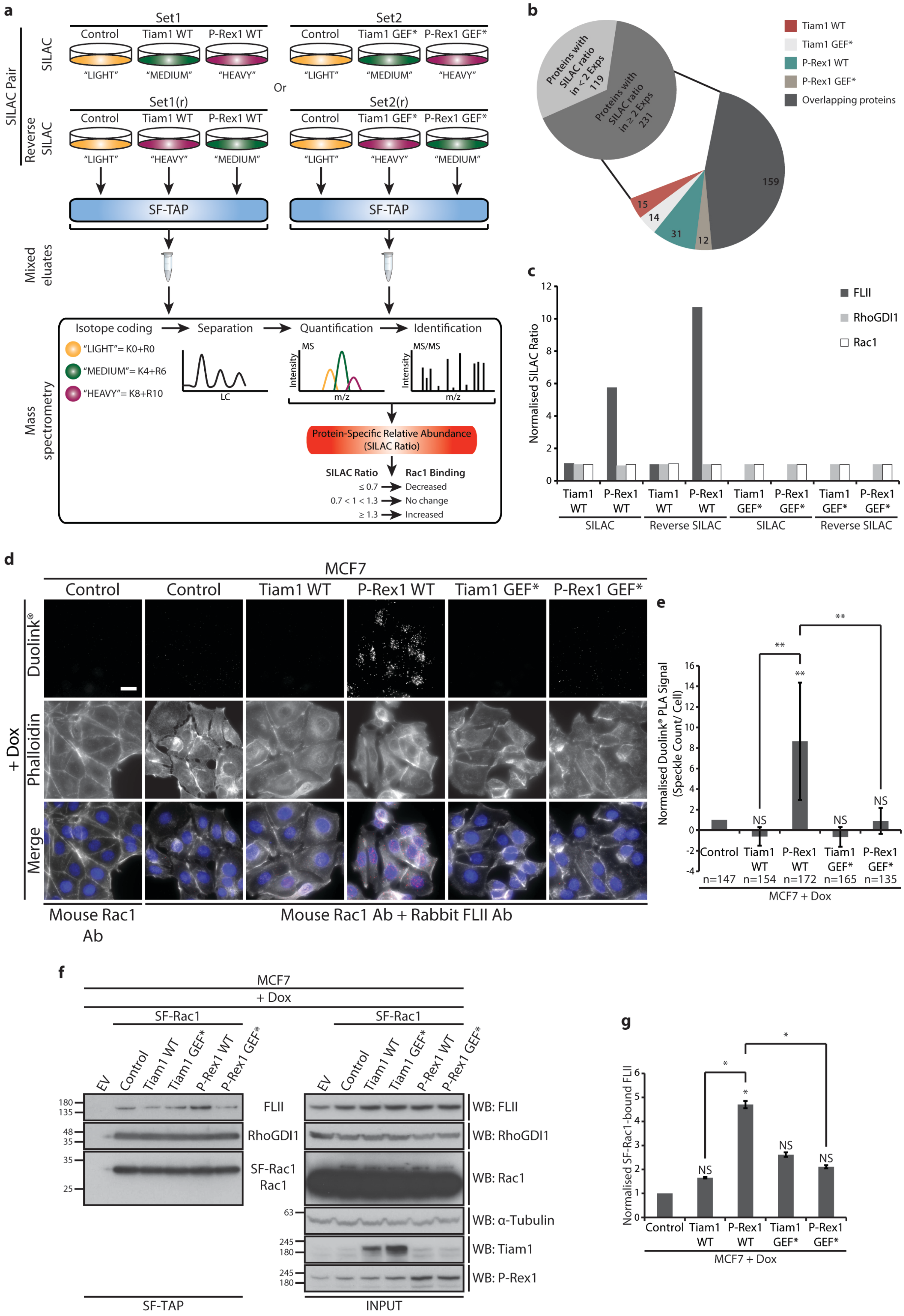

Supplementary Figure 3. Tiam1 and P-Rex1 differentially regulate the Rac1 interactome. (a) Schematic representation of five-way Stable Isotope Labelling by Amino Acid in Cell Culture (SILAC) combined with *Strep*-FLAG Tandem Affinity Purification (SF-TAP). NIH3T3 cells transduced with the doxycycline (dox)-inducible expression system for SF-Rac1 alone (control) or together with Tiam1/P-Rex1 WT (Set1) or GEF* (Set2) were cultured in media containing Lysine (K) and Arginine (R) isotopes as outlined. In reverse SILAC (r) media labels were swapped as indicated. Following dox induction, lysates were subjected to SF-TAP and mixed eluates were analysed by mass spectrometry to identify Rac1 interactors and their abundance calculated as SILAC ratios relative to control cells. A cut off of ± 1.3 fold-change was utilised to infer differential Rac1 binding. **(b)** Pie chart of total identified Rac1 interactors showing number of proteins with GEF-specific changes in Rac1 binding for indicated GEFs. **(c)** Graph representing normalised SILAC ratios for FLII, RhoGDI1 and Rac1 for indicated GEFs from representative SILAC and reverse SILAC screens. **(d)** Representative immunofluorescence images of MCF7 cells fixed in 4 % formaldehyde and subjected to the Duolink® *In Situ* PLA assay following treatment with 1 μg ml $^{-1}$ dox (+ dox) for 24 hours to induce expression of indicated GEF constructs. Phalloidin and DAPI were used to visualise the actin cytoskeleton and nuclei respectively. Scale bar= 20 μm. **(e)** Quantification of average Duolink® PLA signal from indicated number of MCF7 cells described in (d) normalised to control cells ± s.e.m. **(f)** SF-TAP of SF-Rac1 from MCF7 cells treated with 1 μg ml $^{-1}$ dox to induce expression of SF Rac1 alone (control) or together with indicated GEFs. Co-precipitated endogenous FLII and RhoGDI1 were detected by Western blot analysis. α-Tubulin was used as a loading control. **(g)** Quantification of endogenous SF-Rac1-bound FLII in MCF7 cells described in (f) normalised to control cells ± s.e.m. from three independent experiments. In (e) and (g) Student's t-test was used to assess significance as indicated on graph. p-values indicated above each bar are relative to + dox treated control cells. NS= non-significant; *= $p \le 0.05$; **= $p \le 0.01$.

Supplementary Figure 4

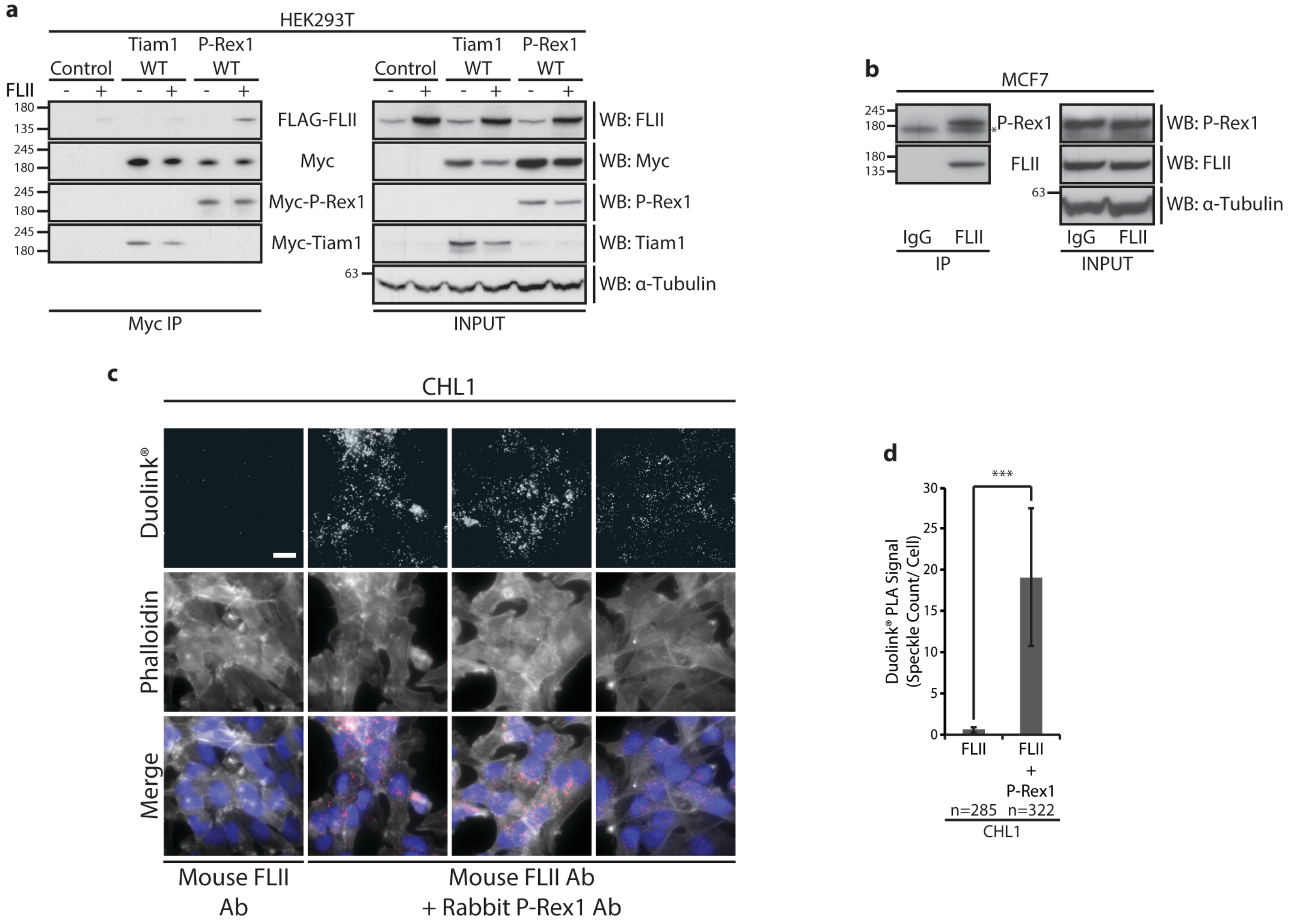

Supplementary Figure 4. FLII binds preferentially to P-Rex1 but not Tiam1. (a) Myc immunoprecipitation (IP) from HEK293T cells expressing Myc-tagged Tiam1 WT or Myc-tagged P-Rex1 WT alone or together with FLAG-tagged FLII. Co-precipitated FLAG-tagged FLII was detected by Western blot analysis. **(b)** Endogenous FLII IP from MCF7 cells. Co-precipitated endogenous P-Rex1 was detected by Western blot analysis. * indicates background band. In (a) and (b) α-Tubulin was used as a loading control. Representative Western blots from three independent experiments. **(c)** Representative immunofluorescence images of CHL1 cells fixed in 4 % formaldehyde and subjected to the Duolink® *In Situ* PLA assay. Phalloidin and DAPI were used to visualise the actin cytoskeleton and nuclei, respectively. Scale bar= 20 µm. **(d)** Quantification of average Duolink® PLA signal from indicated number of CHL1 cells described in (c) ± s.e.m. Student's t-test was performed to determine statistical significance and p-values are shown on graph. ***= $p \leq 0.001$.

Supplementary Figure 5

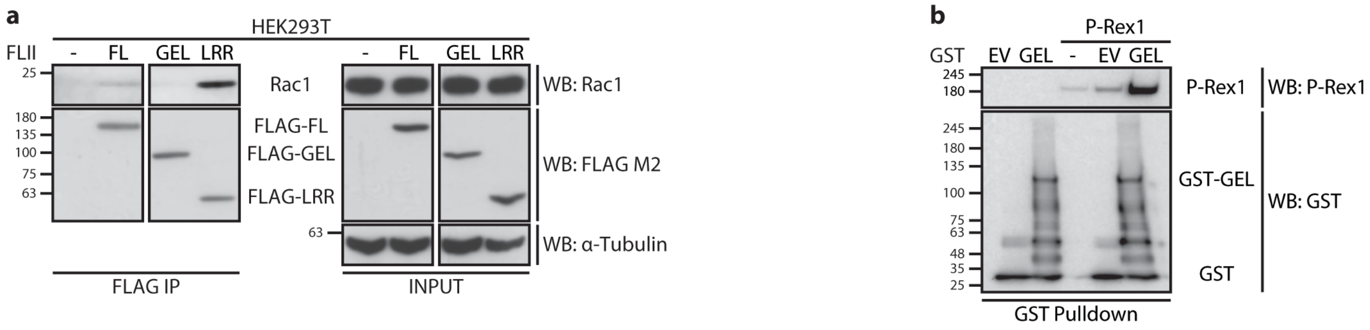

Supplementary Figure 5. Rac1-FLII and P-Rex1-FLII interactions are mediated by different FLII domains.

(a) FLAG immunoprecipitation (IP) from HEK293T cells expressing the different FLAG-tagged FLII domain mutants. Co-precipitated endogenous Rac1 was detected by Western blot analysis. FLII FL= full length FLII; FLII GEL= Gelsolin domain only; FLII LRR= LRR domain only. α-Tubulin was used as a loading control. Representative Western blot from three independent experiments. **(b)** GST pulldown using purified GST (EV) or GST-tagged FLII gelsolin (GEL) domain incubated with full length P-Rex1 recombinant protein. *In vitro* P-Rex1-FLII interaction was detected by Western blot analysis. Representative Western blot from three independent experiments.

Supplementary Figure 6

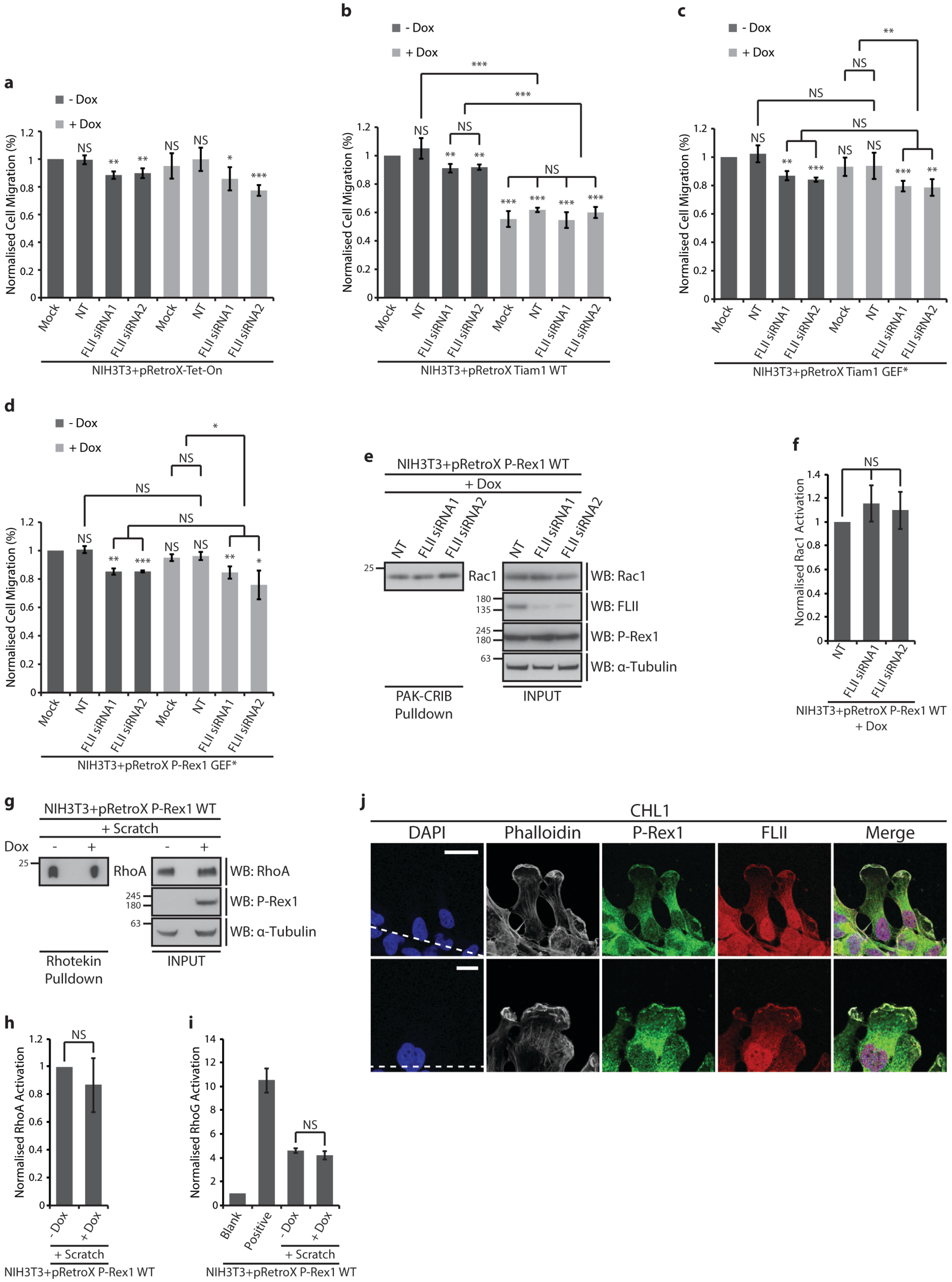

Supplementary Figure 6. FLII is required for optimal cell migration. (a-d) Quantification of NIH3T3 cell migration following treatment with mock, non-targeting (NT) or two different siRNAs against FLII (FLII siRNA1 and FLII siRNA2) together with ethanol (- dox) or 1 $\mu g\ ml^{-1}$ doxycycline (+ dox) to induce expression of indicated GEF constructs. Graphs represent the average percent migration normalised to - dox treated mock cells ± s.e.m from three independent ORIS™ migrations assays. **(e)** Lysates from NIH3T3 cells expressing P-Rex1 WT following dox induction and treatment with indicated oligos were subjected to PAK-CRIB pulldown and levels of active and total Rac1 were detected by Western blot analysis. **(f)** Quantification of active Rac1 levels in NIH3T3 cells described in (e) normalised to NT control ± s.e.m. from three independent experiments. **(g)** NIH3T3 cells were treated with ethanol (- dox) or 1 $\mu g\ ml^{-1}$ dox (+ dox) to induce P-Rex1 WT expression and subjected to multiple scratches to stimulate migration. Levels of active and total RhoA were detected by Western blot analysis following Rhotekin pulldown. **(h)** Quantification of active RhoA levels in NIH3T3 cells described in (g) normalised to NT control ± s.e.m. from three independent experiments. **(i)** Lysates from NIH3T3 cells expressing P-Rex1 WT treated as described in (g) were analysed using an active RhoG ELISA kit. Graph represents average optical density measurements at 450 nm corresponding to active RhoG levels ± s.e.m. from three independent experiments. For (a-d), (f), (h) and (i) Student's t-test was performed to determine statistical significance and p-values are shown on graph. p-values indicated above each bar are relative to – dox treated mock cells (a-d). NS= non-significant; *= $p \leq 0.05$; **= $p \leq 0.01$; ***= $p \leq 0.001$. **(j)** Representative immunofluorescence images of CHL1 cells fixed in 4 % formaldehyde 24 hours post scratching to stimulate cell migration. Phalloidin staining was used to visualise the actin cytoskeleton and antibodies against endogenous P-Rex1 and FLII were used to determine their co-localisation. DAPI was used to stain the nuclei. Scale bar= 20 µm. White dashed lines indicate the position and orientation of the scratch.

Supplementary Figure 7

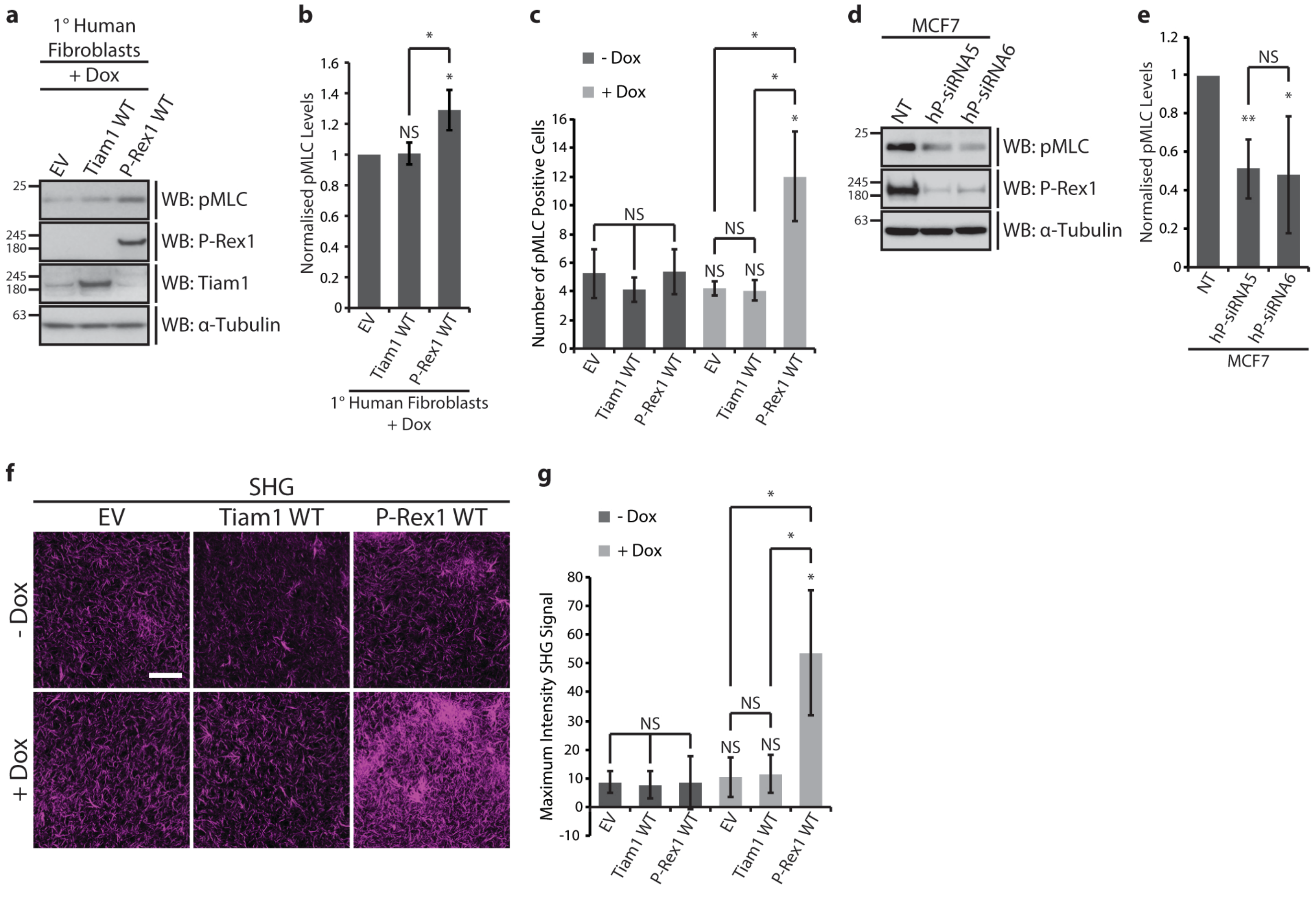

Supplementary Figure 7. P-Rex1 regulates cell contraction. (a) Primary (1°) human fibroblasts were treated with 1 $\mu g\ ml^{-1}$ doxycycline (dox) to induce expression of indicated constructs. Levels of endogenous pMLC were detected by Western blot analysis. α-Tubulin was used as a loading control. **(b)** Quantification of pMLC levels in 1° human fibroblasts described in (a) normalised to α-Tubulin and pMLC levels detected in the empty vector (EV) control. Graph represents average pMLC levels ± s.e.m. from three independent experiments. **(c)** Fibroblast-collagen matrices were treated with ethanol (- dox) or 1 $\mu g\ ml^{-1}$ dox (+ dox) to induce expression of indicated constructs prior to pMLC staining by immunohistochemistry. Graph represents number of pMLC positive cells in fibroblast-collagen matrices ± s.e.m. from three independent experiments. **(d)** MCF7 cells were transfected with non-targeting (NT), or two different siRNAs against human P-Rex1 (hP-siRNA5, hP-siRNA6). Levels of endogenous pMLC and P-Rex1 were detected by Western blot analysis. **(e)** Quantification of pMLC levels in MCF7 cells described in (d) normalised to α-Tubulin and pMLC levels detected in the NT control. Graph represents average pMLC levels ± s.e.m. from three independent experiments. **(f)** Second Harmonic Generation (SHG) imaging of fibroblast-collagen matrices treated as described in (c) showing representative maximum intensity projections of 3D stacked SHG imaging (purple= collagen). Scale bar= 50 μm. **(g)** Quantification of SHG signal intensity from fibroblast-collagen matrices treated as described in (c). Graph represents the intensity

of SHG signal produced by fibrillar collagen ± s.e.m. from three independent experiments. For (b), (c), (e), and (g) Student's t-test was used to assess significance as indicated on graph. p-values indicated above each bar are relative to + dox treated EV cells (b), - dox treated EV control (c, g) or to the NT control (e). NS= non-significant; *= $p \leq 0.05$; **= $p \leq 0.01$.

Supplementary Figure 8

Figure 1b

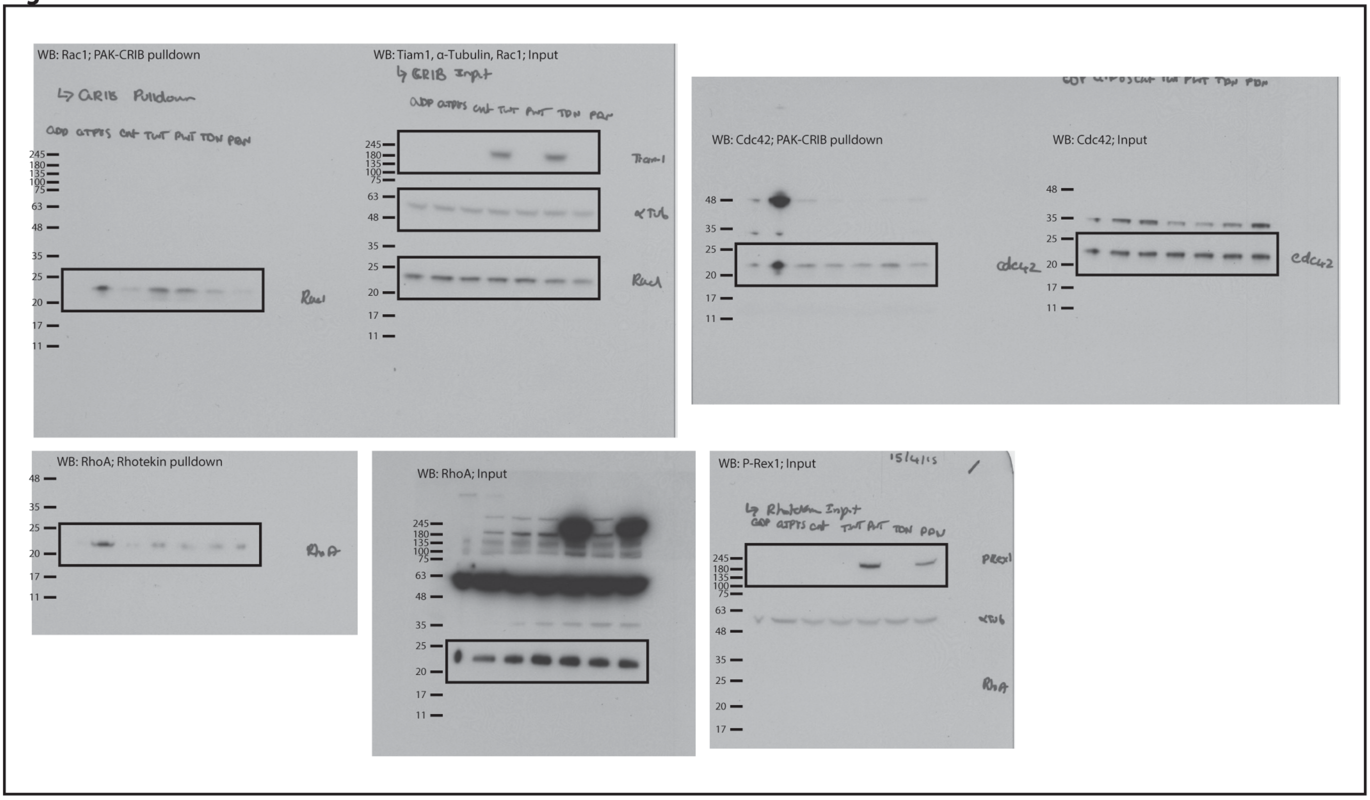

Figure 2f

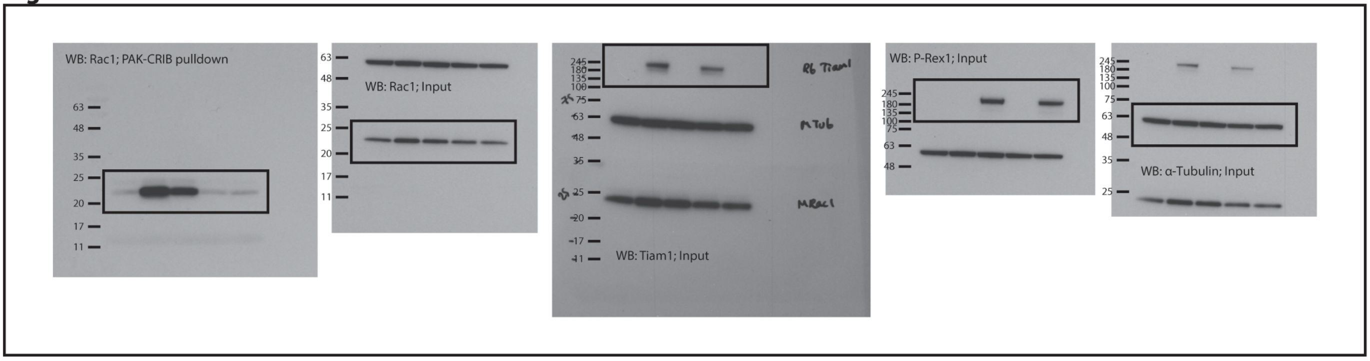

Figure 3b

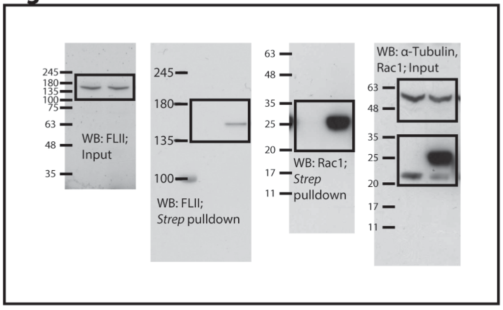

Figure 3c

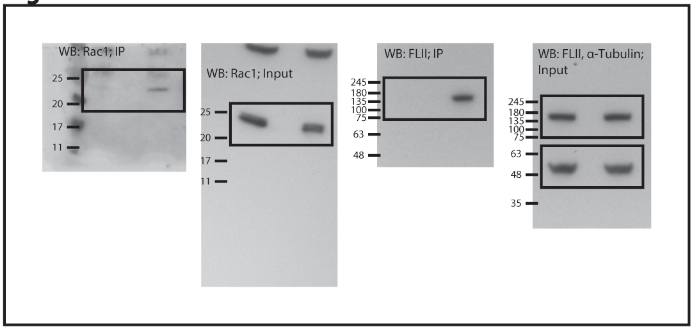

Figure 3d

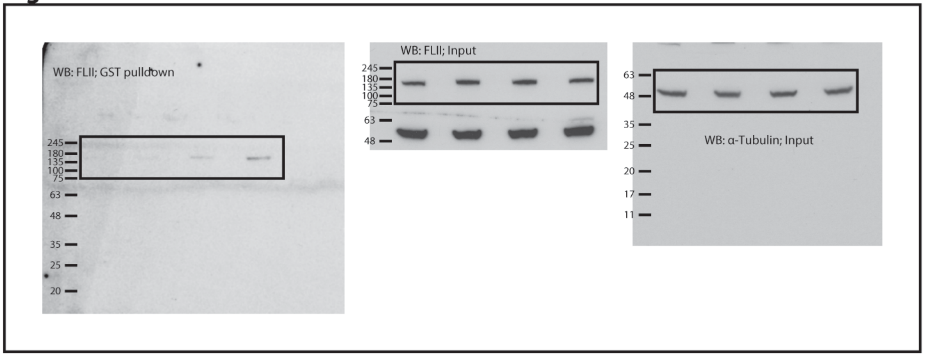

Supplementary Figure 8. Full blot scans for cropped blots presented in Figs. 1, 2 and 3.

Supplementary Figure 9

Figure 4a

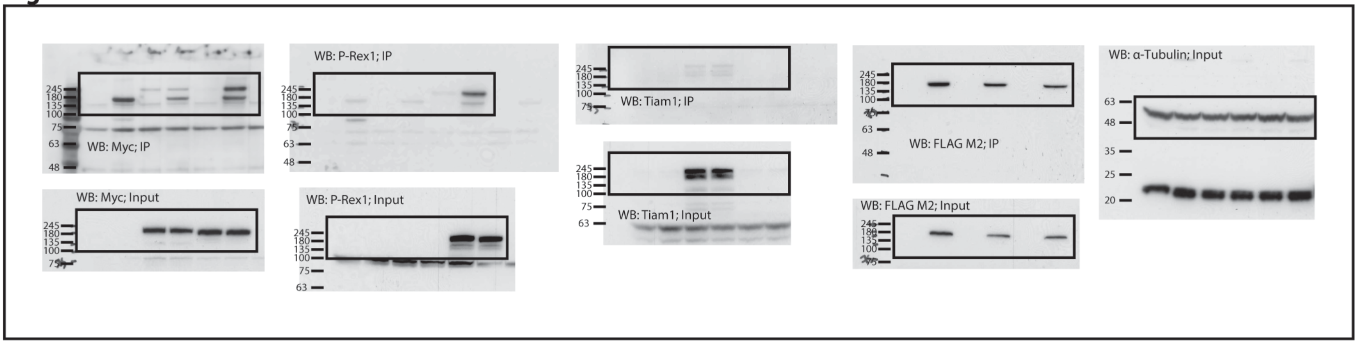

Figure 4b

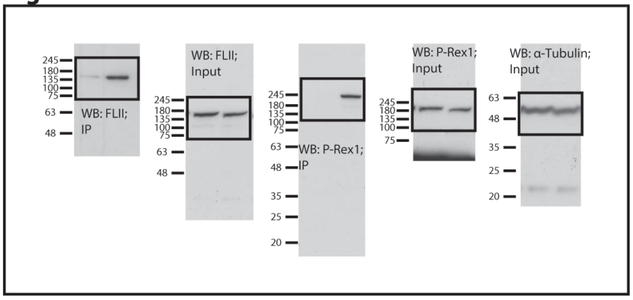

Figure 4c

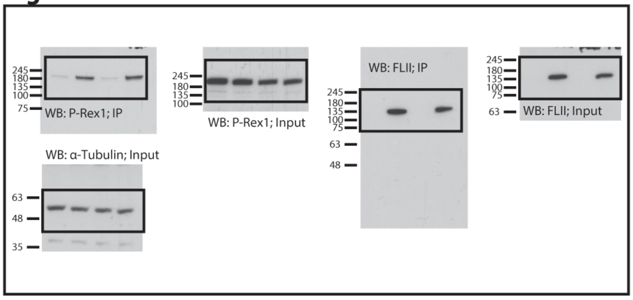

Figure 5b

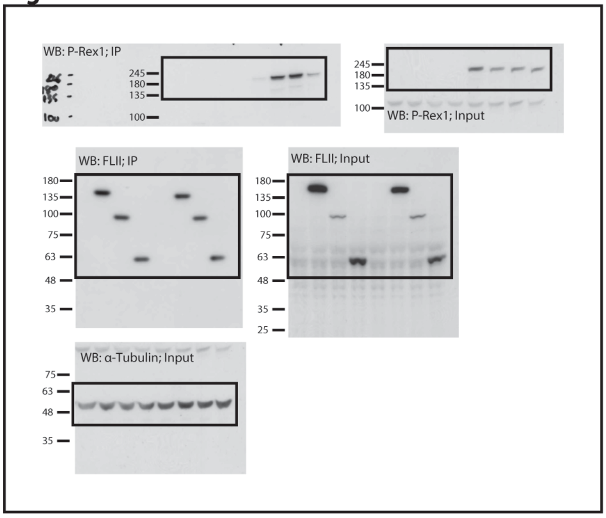

Figure 5c

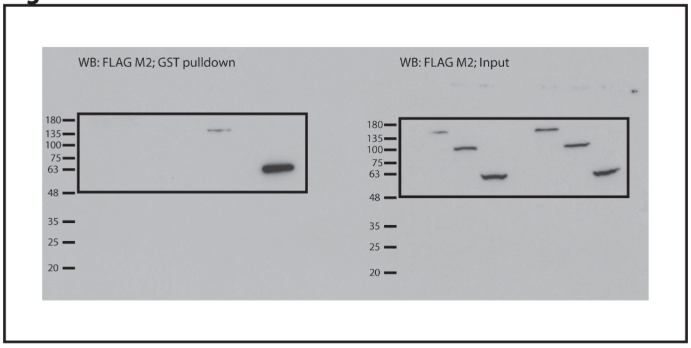

Figure 6a

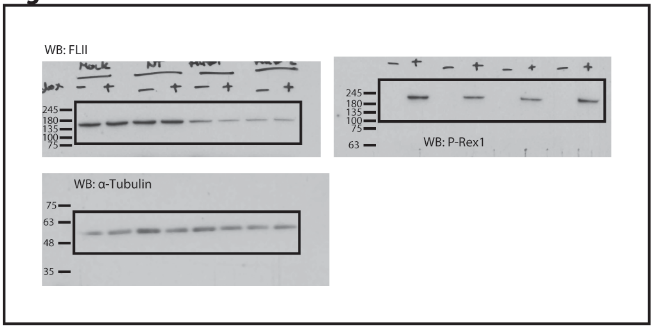

Figure 6h

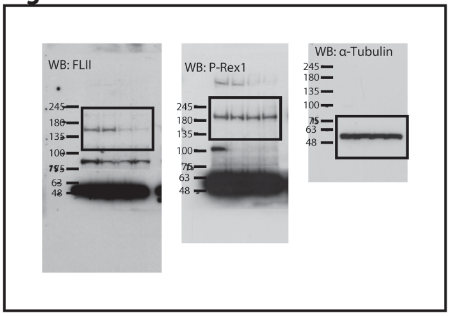

Figure 7c

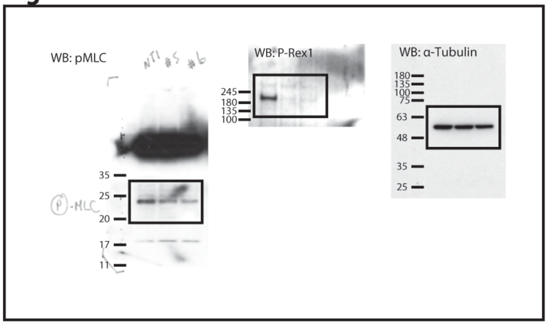

Figure 7j

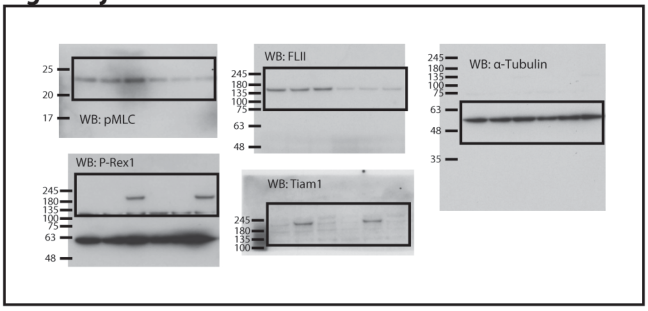

Supplementary Figure 9. Full blot scans for cropped blots presented in Figs. 4, 5, 6 and 7.

Supplementary Table 1. Summary of known, predicted and Rac1 binding proteins implicated in Rac1 signalling identified from SILAC screens.

	Protein Name	Reference
Known Rac1 regulators	Dedicator of cytokinesis protein 7	[1]
	Rho GDP-dissociation inhibitor 1	HPRD [2]
	Rho GDP-dissociation inhibitor 2	HPRD [2]
	Rho GDP-dissociation inhibitor 3	HPRD [2]
Known Rac1 effectors	Alpha-actinin 1	HPRD [2]
	Alpha-tubulin 1	HPRD [2]
	Calmodulin	[3]
	Filamin-A	HPRD [2]
	Filamin-B	[4]
	Ras GTPase-activating-like protein IQGAP1	NetPath [5]
	Ras GTPase-activating-like protein IQGAP2	HPRD [2]
	Ras GTPase-activating-like protein IQGAP3	[6]
Predicted Rac1 binding partners	Alpha-actinin 4	PIPs [7, 8]
	14-3-3 protein gamma	
	14-3-3 protein theta	
	AP-2 complex subunit mu	
	Bifunctional aminoacyl-tRNA synthetase	
	Centaurin beta 2	
	Coatomer subunit epsilon	
	Cofilin-1	
	Dynactin subunit 1	
	E3 ubiquitin-protein ligase HUWE1	
	Heterogeneous nuclear ribonucleoprotein K	
	Peptidyl-prolyl cis-trans isomerase A	
	S-methyl-5-thioadenosine phosphorylase	
	Spectrin alpha 2	
	T-complex protein 1 subunit delta	
	T-complex protein 1 subunit zeta	
Proteins implicated in Rac1 signalling	Actin-related protein 2/3 complex subunit 1B	[9]
	Actin-related protein 2/3 complex subunit 2	[9]
	Actin-related protein 2/3 complex subunit 3	[9]
	Actin-related protein 2/3 complex subunit 4	[9]
	Actin-related protein 3	[9]
	Alpha-actinin 1	[10]
	Cell division control protein 42 homolog	[11, 12]
	Diaphanous-related formin-1	[13]
	Kinesin-1 heavy chain	[14]
	Myosin heavy chain 10	[15]
	Myosin heavy chain 14	[15]
	Myosin heavy chain 9	[15]
	Myosin light chain 3	[15]
	Myosin light polypeptide 6	[15]
	Myosin regulatory light chain 12B	[15]
	Myosin-Ic	[15]
	Myosin-Id	[15]
	Protein flightless-1 homolog	[16]
	Ras-related C3 botulinum toxin substrate 3	[17]
	Zyxin	[18]

Supplementary Table 2. List of primary and secondary antibodies utilised in this study.

Antibody	Species	Manufacturer	Dilution/Remarks
**List of antibodies used for Western blot analysis**			
Anti-HA	Rabbit	Abcam, ab13834	1:5000 of 1 mg ml $^{-1}$
Anti-Mouse-HRP	N/A	GE Healthcare, RPN4201	1:5000
Anti-Rabbit-HRP	N/A	GE Healthcare, RPN4301	1:5000
c-Myc (9E10)	Mouse	Santa Cruz, sc-40	1:1000 of 200 μg ml $^{-1}$
Cdc42 (P1)	Rabbit	Santa Cruz, Sc-87	1:1000 of 200 μg ml $^{-1}$
FLAG® M2	Mouse	Sigma-Aldrich, F1804	1:5000 of 1 mg ml $^{-1}$
Flightless I (116.40)	Mouse	Santa Cruz, sc-21716	1:1000 of 200 μg ml $^{-1}$
FLII	Rabbit	Sigma-Aldrich, HPA007084	1:1000 of 100 μg ml $^{-1}$
P-Rex1	Rabbit	Sigma-Aldrich, HPA001927	1:1000 of 200 μg ml $^{-1}$
P-Rex1	Goat	Sigma-Aldrich, SAB2501302	1:1000 of 500 μg ml $^{-1}$
Phospho-Myosin Light Chain 2 (Ser19)	Rabbit	Cell Signalling, 3671	1:1000 overnight
Rac1 (102)	Mouse	BD Biosciences, 610650	1:1000 of 250 μg ml $^{-1}$
Rac1 (23A8)	Mouse	Millipore, 05-389	1:1000 of 1 mg ml $^{-1}$
RhoA (26C4)	Mouse	Santa Cruz, sc-418	1: 500 of 200 μg ml $^{-1}$
RhoGDI1 (FL-204)	Rabbit	Santa Cruz, sc-33201	1:1000 of 200 μg ml $^{-1}$
Tiam1 (C16)	Rabbit	Santa Cruz, sc-872	1:1000 of 200 μg ml $^{-1}$l
Tiam1	Rabbit	Bethyl, A300-099A	1:1000 of 1 mg ml $^{-1}$
α-Tubulin (DM1A)	Mouse	Sigma-Aldrich, T9026	1:5000 of 1 mg ml $^{-1}$
**List of antibodies/conjugated beads used for immunoprecipitation and pulldowns**			
Anti-FLAG® M2 affinity gel	N/A	Sigma-Aldrich, A2220	50 μl per sample
c-Myc (9E10)	Mouse	Santa Cruz, sc-40	12 μg per sample
Flightless I (116.40)	Rabbit	Santa Cruz, sc-21716	2-4 μg per sample
FLII	Mouse	Sigma-Aldrich, HPA007084	2-4 μg per sample
P-Rex1	Rabbit	Sigma-Aldrich, HPA001927	2-4 μg per sample
P-Rex1	Goat	Sigma-Aldrich, SAB2501302	2-4 μg per sample
Rac1 (102)	Mouse	BD Biosciences, 610650	2 μg per sample
Strep-Tactin® superflow resin	N/A	(IBA GmbH, 2-1206-10),	60-200 μl per sample
**List of antibodies used for immunofluorescence**			
Alexa Fluor® 568 Phalloidin	N/A	Life Technologies, A12380	1:100
Anti-HA	Rabbit	Abcam, ab13834	1:500
E-cadherin, DECMA-1	Rat	Abcam, ab11512	1:100
Myc-488 conjugated	N/A	Life Technologies, 13-2511	1:100
Secondary Alexa Flour® conjugated antibodies (488 & 568)	N/A	Life Technologies	1:500

Supplementary Table 3. Details of constructs utilised in this study.

Construct Label	Vector Backbone	Insert	Supplier/Cloning Strategy
FLAG-FLII-FL	pcDNA3.1	FLAG-FLII full length	Kind gift from the Stallcup group, University of Southern California, USA. Previously described by Lee et al. [19].
FLAG-FLII-GEL	pSG5	FLAG-FLII GEL domain	
FLAG-FLII-LRR	pSG5	FLAG-FLII LRR domain	
pBOS-Histone-2B-GFP	pBOS	Histone-2B-GFP	Kind gift from the Lacaud group, CRUKMI, UK. Previously described by Woodcock et al. [20].
pRetroX-Tiam1 GEF*	pRetroX-Tight-Pur	HA-Tiam1 GEF*	The Malliri group, CRUKMI, UK. Previously described by Mack et al. [21].
pRetroX-Tiam1 WT	pRetroX-Tight-Pur	HA-Tiam1 WT	
PLHCX-P-REX1 WT	PLHCX	MYC-P-REX1 WT	Kind gift from the Welch group, Babraham Institute, University of Cambridge, UK. Previously described by Lindsay et al. [22].
pLHCX-P-Rex1 GEF*	pLHCX	Myc-P-Rex1 GEF*	
pRetroX-P-Rex1 WT	pRetroX-Tight-Pur	Myc-P-Rex1 WT	Made in-house by subcloning Myc-P-Rex1 WT from pLHCX-P-Rex1 WT following PCR amplification and introduction of XbaI and MluI restriction sites using the following primers:<br>Forward primer: Xba1 sequence in bold<br>5'-acct**tctaga**accatggagcagaagctgatc-3'<br>Reverse primer: Mlu1 sequence in bold<br>5'-acct**acgcgtt**Tcagaggtccccatccsccgg-3'
pRetroX-P-Rex1 GEF*	pRetroX-Tight-Pur	Myc-P-Rex1 GEF*	Made in-house by subcloning the first 3.5 kb of Myc-P-Rex1 GEF* from pLHCX-P-Rex1 GEF* following PCR amplification and introduction of XbaI restriction site using the following primers:<br>Forward primer: Xba1 sequence in bold<br>5'-acct**tctaga**accatggagcagaagctgatc-3'<br>Reverse primer:<br>5'-gagtggggataactcatggt-3'
pcDNA3-Myc Tiam1 WT	pcDNA3.0	Myc-Tiam1 WT	Kind gift from the Der Group, University of North Carolina. Previously described by Lambert et al. [23]
N-SF-TAP-pcDNA3	pcDNA3.0	N-terminal SF-tag	Kind gift from the Ueffing group, University of Tuebingen, Germany. Previously described by Gloeckner et al. [24].
pRetroX-Tet-On Advanced	N/A	N/A	Clontech, PT3968-5; 632104.
pRetroX-Tight-Pur	N/A	N/A	Clontech, PT3960-5; 632104/632105.
pcDNA3-Myc Rac1 WT	pcDNA3.0	Myc-Rac1 WT	The Malliri group, CRUMI, UK. Previously described by Matos et al. [25].
pcDNA3-SF-Rac1 WT	pcDNA3.0	SF-Rac1 WT	Made in-house by subcloning Rac1 WT (without the Myc tag) from pcDNA3-Myc Rac1 WT following PCR amplification and introduction of NheI and XbaI restriction sites using the following primers:<br>Forward primer: NheI sequence in bold<br>5'-T**GCTAGC**CTGCAGGCCATCAA-3'<br>Reverse primer: XbaI sequence in bold<br>5'-ACTG**TCTAGA**GGATCCTTACAACACAGGCA-3'
pRetroX-SF-Rac1	pRetroX-Tight-Pur	SF-Rac1 WT	Made in-house by subcloning SF-Rac1 WT from pcDNA3-SF-Rac1 WT using BamHI restriction site.
pGEX-GST	pGEX-4T-1	GST	Kind gift from the Burridge group, University of North Carolina, USA. Previously described by Garcia-Mata et al. [26]
pGEX-GST-Rac1 WT	pGEX-4T-1	GST-Rac1 WT	
pGEX-GST-FLII GEL	pGEX-4T-1	GST-FLII Gelsolin	Kind gift from the McCulloch group, University of Toronto, Canada. Previously described by Arora et al. [27] and Mohammad et al. [28].

Supplementary Table 4. SILAC Amino Acid Isotope Stock Solutions.

SILAC Stock Solution[1]	Amino Acid Isotope
K0 stock solution [2]	L-Lysine monohydrochloride (Sigma-Aldrich, L5626)
K4 stock solution [2]	4.4.5.5-D4-L-Lysine HCL (Silantes, 211104112)
K8 stock solution [2]	L-Lysine HCL ^{13}C, ^{15}N (Silantes, 211604102)
R0 stock solution [3]	L-Arginine monohydrochloride (Sigma-Aldrich, A5131)
R6 stock solution [3]	L-Arginine HCL ^{13}C (Silantes, 201204102)
R10 stock solution [3]	L-Arginine HCL ^{13}C, ^{15}N (Silantes, 201604102)

[1] Stock solutions were stored in 1 ml aliquots at 4 °C
[2] Lysine (K) final concentration of 146 mg ml^{-1} in PBS
[3] Arginine (R) final concentration of 84 mg ml^{-1} in PBS

Supplementary Table 5. NIH3T3 SILAC Labelling.

SILAC Experiment	SILAC Media	Isotope Composition	NIH3T3 GEF Expression
**SILAC**	LIGHT	K0+R0	Control (SF-Rac1 only)
	MEDIUM	K4+R6	Tiam1 WT; Tiam1 GEF*
	HEAVY	K8+R10	P-Rex1 WT; P-Rex1 GEF*
**Reverse SILAC**	LIGHT	K0+R0	Control (SF-Rac1 only)
	MEDIUM	K4+R6	P-Rex1 WT; P-Rex1 GEF*
	HEAVY	K8+R10	Tiam1 WT; Tiam1 GEF*

Supplementary Methods

Analysis of cell morphology. For NIH3T3 cells, given their mesenchymal phenotype, the three groups used for assessing GEF-induced changes in cell morphology were as follows: 1) mesenchymal, 2) epithelial-like and 3) elongated. In brief, NIH3T3 cells with the normal triangular fibroblast morphology were considered mesenchymal. Cells exhibiting increased cell aggregation accompanied by membrane ruffling were considered as epithelial-like, while cells with increased numbers of thin membrane protrusions as elongated. In contrast, A431 and MDCKII cell lines have a characteristic epithelial morphology, thus the three groups used for morphological classification of these cells were as follows: 1) epithelial, 2) compact epithelial-like and 3) elongated. Given that both the first and second groups entailed an epithelial phenotype, to distinguish between the two morphologies, more emphasis was put on how compact the colonies were. Thus the compact epithelial-like morphology was assigned to compact colonies with cells harbouring more defined cell junctions with or without membrane ruffling. Cells with a mesenchymal-like phenotype and increased numbers of membrane protrusions were assigned as elongated. For all cell lines investigated phase-contrast images were captured from three independent experiments and a total of 150 cells were analysed per condition. Graphs represent number of cells within each category as a percentage of the total number of cells analysed.

Cell scattering assays. To quantify cell scattering, a total of 50 colonies per condition were examined and classified into unscattered or scattered depending on the morphology of cells within a colony. Colonies with more than 3 cells exhibiting a mesenchymal-like morphology or reduced cell-cell contacts with neighbouring cells were considered scattered. It is important to note that single cells were not included for this assay and that only colonies with ≥ 3 cells were analysed. Graphs represent number of colonies identified in each group as a percentage of total number of colonies analysed from three independent experiments.

ORIS™ migration assay. To account for variability in cell migration 3-8 wells were used as technical replicas for each condition. The effects of GEF expression on cell migration was evaluated by calculating the percent migration using the following equation:

$$\frac{(\text{24 hours migration})_{\text{Area}} - (\text{0 hours migration})_{\text{Area}}}{(\text{0 hour migration})_{\text{Area}}} \text{ X } 100$$

Percent migration values obtained for each sample were normalised to the appropriate control described in the figure legends. Graphs represent normalised average percent migration from three independent experiments.

Single cell tracking. For live-imaging of wound closure, cells were imaged using the Opera Phenix microscope (Perkin Elmer) with temperature and environmental controls (37°C and 5 % CO_2) using the 40x 1.1 NA water immersion lens (Zeiss). Frames were captured at 20 minutes intervals for 15 hours. Multi-plane TIFs were exported from the Columbus software and analysed manually in Image J, using the manual tracking plugin to track cell migration into the wound space over time.

Fibroblast-collagen matrix contraction assay. To account for variability in cell contraction at least 3 matrices were used as technical replicas for each condition. Collagen gels were scanned using an EPSON PERFECTION 3200 PHOTO scanner at 0 and 24 hours post ethanol or doxycycline (dox) treatment and the percent of fibroblast-collagen matrix contraction was calculated using the following equation:

$$\frac{\text{(Matrix surface area at 24 hours) - (Matrix surface area at 0 hours)}}{\text{(Matrix surface area at 0 hours)}} \text{ X } 100$$

Percent fibroblast-collagen matrix contraction values obtained for each sample were normalised to the appropriate control described in the figure legends. Graphs represent normalised average percent fibroblast-collagen matrix contraction from three independent experiments.

Second harmonic generation imaging of fibrillar collagen I. Acquisition was performed on Leica SP8 inverted multiphoton microscope, using a 20x magnification and 0.95 NA water objective. The multiphoton laser source was a Ti:Sapphire femto-second laser cavity (Coherent Chameleon Ultra II), coupled into a LaVision Biotec Trim-scope scan-head and tuned to an excitation wavelength of 840 nm. Second harmonic generation (SHG) signals were collected on a nondescanned external hybrid detector and a 420/40-nm emission filter was used. A 20 µm z-stack with 1.5 µm step size was imaged over a region of 512 µm X 512 µm, with 3 regions of interest per sample taken. The intensity of the SHG signal was calculated using ImageJ software.

Gray-level co-occurrence matrix analysis. First, a directory containing the maximum projections derived from SHG z-stacks was selected for each sample. Then the GLCM plugin (UMB GLCM features [29]) compared 5 texture parameters (contrast, uniformity, correlation, homogeneity and entropy) in numerous directions and distances and a GLCM matrix was calculated for each image. Once all the images in the directory were analysed, the mean (with s.e.m.) value of each texture parameter for each image was calculated using a MATLAB script (MathWorks, Chatswood, Australia). The results of the GLCM correlation were then fitted to a double exponential decay model in GraphPad, Prism software (GraphPad Software, La Jolla, CA, USA). Graphs represent the GLCM correlation decay curves calculated for – dox and + dox treated fibroblast-collagen matrices. Slower decay indicates a denser collagen ultrastructure than samples with a faster decay.

Microscopy. Phase-contrast microscopy was performed on a Zeiss Axiovert 2.5 using a CP-ACROMAT 10x/0.25 magnification lens. Images were captured with a Canon camera (DS126191) and processed using the Axiovision Release 4.8 software (Zeiss). For immunofluorescence, images were taken on the Low Light microscope system based on a Zeiss Axiovert 200M enclosed in a full environmental chamber (Solent Scientific). The system utilises Metamorph software and - 80 °C cooled Roper Cascade EMCCD 512B back illuminated camera. For fluorescence illumination a 300 W Xenon light source was used. Additionally, wavelength switching was automated using shutters and filter wheels with the ET-Sedat filter set (406/488/568/647 nm excitation). Data was imaged with Zeiss alpha plan flour 40x objective lens or 100x 1.45 NA oil immersion objective lens. Co-localisation images were captured using the Gated Stimulated Emission Depletion (gSTED) microscope system. Additional imaging systems utilised in this study are indicated in the appropriate functional assay sections.

Duolink® *In Situ* Proximity Ligation Assay. For quantification of the Duolink® PLA signal, images were imported into the CellProfiler software to count the number of speckles per image, which was then normalised to the number of nuclei in the field of view to obtain the number of speckles per cell. To account for background, control samples were prepared for each experiment in which cells were only incubated with one primary antibody followed by the Duolink® *In Situ* PLA protocol. Speckle counts per cell from these samples were subtracted from the average speckle count per cell identified for each condition. The obtained numbers were normalised to the appropriate control described in the figure legends. For detecting P-Rex1-FLII endogenous binding in CHL1 cells, the single antibody control signal was not subtracted from samples

subjected to both anti-P-Rex1 and anti-FLII antibodies and was plotted as a background signal reference instead.

Purification of GST, GST-Rac1 WT and GST-FLII GEL. Glycerol stocks of pGEX-GST, pGEX-GST Rac1 WT and pGEX-GST FLII GEL transfected Rosetta™ 2 competent cells were plated overnight on L-agar + ampicillin. Single colonies were picked and cultured overnight in 10 ml LB + ampicillin at 37 °C, then added to 400 ml LB + ampicillin at 30 °C. Once the optical density of cultures was between 0.6-0.8 at 600 nm, protein expression was induced by adding IPTG (100 mM; Sigma-Aldrich, I5502) overnight at 24 °C. Cultures were then harvested, pelleted and resuspended in resuspension buffer [0.5 M NaCl, 1 mM EDTA, protease inhibitor cocktail tablet (Roche, 11697498001) in PBS] prior to sonication (3x 1 minute at 10 Amp), addition of 500 μl of 20 % Triton-x-100 and pelleting for 120 minutes. The supernatant was then incubated with 500 μl pre-washed Glutathione Sepharose™4B for 2 hours at 4 °C, washed, resuspended in 20 % glycerol and stored as aliquots at – 80 °C. for the *in vitro* protein interaction experiments, GST and GST-FLII GEL were incubated with elution buffer [1mM EGTA pH 8.0, 100 mM KCl, 50 mM Tris-HCl pH 8.0, 0.5 mM DTT, 20 mM reduced Glutathione (GSH), and 1mM PMSF in dH_2O] to elute proteins from beads. Prior to experiment, proteins were washed in GST lysis buffer [25 mM Tris-HCl pH 7.2, 150 mM NaCl, 5mM $MgCl_2$, 1 % Nonidet P40 (v/v), 5% glycerol (v/v), 1 % protease inhibitor cocktail (v/v), 1 % phosphatase inhibitor cocktails 1 and 2 (v/v) in dH_2O] and incubated for 1 hour at 4 °C with pre-washed Glutathione Sepharose™4B beads.

Stable Isotope Labelling by Amino Acid in Cell Culture (SILAC)

SILAC labelling

Amino acid stock solutions used to prepare the SILAC labelling media are outlined in Supplementary Table 4. The different SILAC labelling media used in the different experiments are detailed in Supplementary Table 5.

SILAC amino acid incorporation check

After six doubling rounds a small batch of cells were lysed and amino acid incorporation was determined by examining the ratio of lysine and arginine labelled protein peptides versus the unlabelled portion using mass spectrometry. The amino acid incorporation rate was then calculated using the equation outlined below:

$$1 - \left[\frac{1}{(\text{Ratio H/L}) + 1} \right]$$

H= Labelled protein peptides

L= Unlabelled protein peptides

Sample preparation

NIH3T3 cells transduced with the dox inducible system for expressing SF-Rac1 alone or together with the different GEF constructs were plated at 2 x 10^7 cells per 500 cm^2 plate with two plates per condition. After 48 hours cells were treated with 1 μg ml^{-1} doxycycline (+ dox) and incubated for an additional 48 hours. Cells were then subjected to SF-TAP. Following the FLAG® peptide elution step, the different SILAC labelled samples were mixed together and concentrated using a 4 ml Amicon filter unit (EMD Millipore, UFC800308). Concentrated mixed SF-TAP eluates were then subjected to mass spectrometry. For this, a third of each sample was resolved on 12 % NuPAGE®Novex® Bis-Tris pre-cast gels for 9 minutes. Gels were then stained with Coomassie Blue to visualise bands and cut as one section for processing.

In-Gel digestion

In-gel digestion was performed as described by Krug et al. [30] with minor modifications. In brief, 10 mM ammonium bicarbonate and acetonitrile (1:1 v/v) were used to destain gel pieces by washing three times while agitating. Proteins' reduction was then achieved by incubating with 10 mM dithiothreitol (DTT) in 20 mM ammonium bicarbonate for 45 minutes at 56 °C. This was followed by incubation with 55 mM iodoacetamide in 20 mM ammonium bicarbonate for 30 minutes at room temperature in the dark to alkylate the protein samples. Proteins were then washed twice with 5 mM ammonium bicarbonate followed by one acetonitrile wash and dehydrated using a vacuum centrifuge. Proteins were then pre-digested with Lys-C (Wako) (12.5 ng μl^{-1} in 20 mM ammonium bicarbonate) and digested with trypsin (Promega). Resultant peptides were then extracted first in three consecutive steps using the following solutions in the stated order: 1) 3 % TFA in 30% acetonitrile; 2) 0.5 % acetic acid in 80 % acetonitrile; 3) 100% acetonitrile. Upon evaporation, the peptide fractions were desalted using StageTips.

Nano-LC-MS/MS analysis

The nano-LC-MS/MS analysis was performed as described by Krug et al. [30] with a few adjustments. In brief, all peptide fractions were measured using the EASY-nLC II nano-LC coupled to an Orbitrab Velos mass spectrometer. Chromatographic separation was conducted on a 15 cm PicoTip fused silica emitter with 75 μM inner diameter and 8 μM Tip inner diameter with reversed-phase ReproSil-Pur C18-AQ 3 μM resin. Together with 0.5 % acetic acid, peptides were injected into the column at 700 nL $minute^{-1}$ using a maximum pressure of 280 Bar. Elution of peptides was then achieved by using a 121 minutes or a 51 minutes segmented gradient of

5-50 % solvent B (80 % acetonitrile in 0.5 % acetic acid) at a flow rate of 200 nL per minute. The mass spectrometer was operated on a data-dependent mode. Using a resolution of 60,000 and a target value of 1E6 charges, survey full-scans for the MS spectra were recorded between 300-2000 Thompson. The upper most intense 15 peaks from the scans were selected for fragmentation using collision-induced dissociation at a target value of 5000 charges. The resultant spectra were then recorded in the linear ion trap. Selected masses were included in a dynamic exclusion list for 90 seconds.

Data processing and analysis

MS raw files were processed using MaxQuant software (v.1.2.2.9) as described by Carpy et al. [31]. Multiplicity was set as three to match the number of SILAC labels used (i.e. LIGHT, MEDIUM, HEAVY) with K4+R6 set as medium and K8+R10 set as heavy. Following peptide quantification, a database search was performed using the Andromeda search engine. For proteome measurements trypsin was set as the enzyme used. Data was searched against the Uniprot mouse reference proteome downloaded on January 12, 2012. For statistical significance between detected SILAC ratios, the MaxQuant software was used to determine the significance B value associated with each identified protein with p-value ≤ 0.05 considered significant.

Supplementary References

1. Watabe-Uchida M, John KA, Janas JA, Newey SE, Van Aelst L. The Rac activator DOCK7 regulates neuronal polarity through local phosphorylation of stathmin/Op18. *Neuron* **51**, 727-739 (2006).

2. Prasad TS, Kandasamy K, Pandey A. Human Protein Reference Database and Human Proteinpedia as discovery tools for systems biology. *Methods Mol Biol* **577**, 67-79 (2009).

3. Vidal-Quadras M, *et al.* Rac1 and calmodulin interactions modulate dynamics of ARF6-dependent endocytosis. *Traffic* **12**, 1879-1896 (2011).

4. Jeon YJ, *et al.* Filamin B serves as a molecular scaffold for type I interferon-induced c-Jun NH2-terminal kinase signaling pathway. *Mol Biol Cell* **19**, 5116-5130 (2008).

5. Kandasamy K, *et al.* NetPath: a public resource of curated signal transduction pathways. *Genome Biol* **11**, R3 (2010).

6. Wang S, *et al.* IQGAP3, a novel effector of Rac1 and Cdc42, regulates neurite outgrowth. *J Cell Sci* **120**, 567-577 (2007).

7. McDowall MD, Scott MS, Barton GJ. PIPs: human protein-protein interaction prediction database. *Nucleic Acids Res* **37**, D651-656 (2009).

8. Scott MS, Barton GJ. Probabilistic prediction and ranking of human protein-protein interactions. *BMC bioinformatics* **8**, 239 (2007).

9. Miki H, Yamaguchi H, Suetsugu S, Takenawa T. IRSp53 is an essential intermediate between Rac and WAVE in the regulation of membrane ruffling. *Nature* **408**, 732-735 (2000).

10. Kovac B, Teo JL, Makela TP, Vallenius T. Assembly of non-contractile dorsal stress fibers requires alpha-actinin-1 and Rac1 in migrating and spreading cells. *J Cell Sci* **126**, 263-273 (2013).

11. Price LS, Leng J, Schwartz MA, Bokoch GM. Activation of Rac and Cdc42 by integrins mediates cell spreading. *Mol Biol Cell* **9**, 1863-1871 (1998).

12. Royal I, Lamarche-Vane N, Lamorte L, Kaibuchi K, Park M. Activation of cdc42, rac, PAK, and rho-kinase in response to hepatocyte growth factor differentially regulates epithelial cell colony spreading and dissociation. *Mol Biol Cell* **11**, 1709-1725 (2000).

13. Lammers M, Meyer S, Kuhlmann D, Wittinghofer A. Specificity of interactions between mDia isoforms and Rho proteins. *J Biol Chem* **283**, 35236-35246 (2008).

14. Takahashi K, Suzuki K. Requirement of kinesin-mediated membrane transport of WAVE2 along microtubules for lamellipodia formation promoted by hepatocyte growth factor. *Exp Cell Res* **314**, 2313-2322 (2008).

15. van Leeuwen FN, van Delft S, Kain HE, van der Kammen RA, Collard JG. Rac regulates phosphorylation of the myosin-II heavy chain, actinomyosin disassembly and cell spreading. *Nat Cell Biol* **1**, 242-248 (1999).

16. Kopecki Z, O'Neill GM, Arkell RM, Cowin AJ. Regulation of focal adhesions by flightless i involves inhibition of paxillin phosphorylation via a Rac1-dependent pathway. *J Invest Dermatol* **131**, 1450-1459 (2011).

17. Vaghi V, *et al.* Rac1 and rac3 GTPases control synergistically the development of cortical and hippocampal GABAergic interneurons. *Cerebral cortex* **24**, 1247-1258 (2014).

18. Sun Z, Huang S, Li Z, Meininger GA. Zyxin is involved in regulation of mechanotransduction in arteriole smooth muscle cells. *Front Physiol* **3**, 472 (2012).

19. Lee YH, Campbell HD, Stallcup MR. Developmentally essential protein flightless I is a nuclear receptor

coactivator with actin binding activity. *Mol Cell Biol* **24**, 2103-2117 (2004).

20. Woodcock SA, *et al.* Tiam1-Rac signaling counteracts Eg5 during bipolar spindle assembly to facilitate chromosome congression. *Curr Biol* **20**, 669-675 (2010).

21. Mack NA, *et al.* beta2-syntrophin and Par-3 promote an apicobasal Rac activity gradient at cell-cell junctions by differentially regulating Tiam1 activity. *Nat Cell Biol* **14**, 1169-1180 (2012).

22. Lindsay CR, *et al.* P-Rex1 is required for efficient melanoblast migration and melanoma metastasis. *Nat Commun* **2**, 555 (2011).

23. Lambert JM, *et al.* Tiam1 mediates Ras activation of Rac by a PI(3)K-independent mechanism. *Nat Cell Biol* **4**, 621-625 (2002).

24. Gloeckner CJ, Boldt K, Schumacher A, Roepman R, Ueffing M. A novel tandem affinity purification strategy for the efficient isolation and characterisation of native protein complexes. *Proteomics* **7**, 4228-4234 (2007).

25. Matos P, Collard JG, Jordan P. Tumor-related alternatively spliced Rac1b is not regulated by Rho-GDP dissociation inhibitors and exhibits selective downstream signaling. *J Biol Chem* **278**, 50442-50448 (2003).

26. Garcia-Mata R, Wennerberg K, Arthur WT, Noren NK, Ellerbroek SM, Burridge K. Analysis of activated GAPs and GEFs in cell lysates. *Methods Enzymol* **406**, 425-437 (2006).

27. Arora PD, Wang Y, Bresnick A, Janmey PA, McCulloch CA. Flightless I interacts with NMMIIA to promote cell extension formation, which enables collagen remodeling. *Mol Biol Cell* **26**, 2279-2297 (2015).

28. Mohammad I, *et al.* Flightless I is a focal adhesion-associated actin-capping protein that regulates cell migration. *FASEB J* **26**, 3260-3272 (2012).

29. Plugins NUfLSNI.

30. Krug K, Carpy A, Behrends G, Matic K, Soares NC, Macek B. Deep coverage of the Escherichia coli proteome enables the assessment of false discovery rates in simple proteogenomic experiments. *Mol Cell Proteomics* **12**, 3420-3430 (2013).

31. Carpy A, *et al.* Absolute proteome and phosphoproteome dynamics during the cell cycle of fission yeast. *Mol Cell Proteomics*, (2014).
